# LED color gradient as a new screening tool for rapid phenotyping of plant responses to light quality

**DOI:** 10.1093/gigascience/giab101

**Published:** 2022-01-27

**Authors:** Pierre Lejeune, Anthony Fratamico, Frédéric Bouché, Samuel Huerga-Fernández, Pierre Tocquin, Claire Périlleux

**Affiliations:** InBioS - PhytoSYSTEMS, Laboratory of Plant Physiology, University of Liège, B22 Sart Tilman Campus, 4 Chemin de la Vallée, B-4000 Liège, Belgium; InBioS - PhytoSYSTEMS, Laboratory of Plant Physiology, University of Liège, B22 Sart Tilman Campus, 4 Chemin de la Vallée, B-4000 Liège, Belgium; InBioS - PhytoSYSTEMS, Laboratory of Plant Physiology, University of Liège, B22 Sart Tilman Campus, 4 Chemin de la Vallée, B-4000 Liège, Belgium; InBioS - PhytoSYSTEMS, Laboratory of Plant Physiology, University of Liège, B22 Sart Tilman Campus, 4 Chemin de la Vallée, B-4000 Liège, Belgium; InBioS - PhytoSYSTEMS, Laboratory of Plant Physiology, University of Liège, B22 Sart Tilman Campus, 4 Chemin de la Vallée, B-4000 Liège, Belgium; InBioS - PhytoSYSTEMS, Laboratory of Plant Physiology, University of Liège, B22 Sart Tilman Campus, 4 Chemin de la Vallée, B-4000 Liège, Belgium

**Keywords:** imaging, phenomics, light gradient, red:blue ratio, controlled environment agriculture

## Abstract

**Background:**

The increasing demand for local food production is fueling high interest in the development of controlled environment agriculture. In particular, LED technology brings energy-saving advantages together with the possibility of manipulating plant phenotypes through light quality control. However, optimizing light quality is required for each cultivated plant and specific purpose.

**Findings:**

This article shows that the combination of LED gradient set-ups with imaging-based non-destructive plant phenotyping constitutes an interesting new screening tool with the potential to improve speed, logistics, and information output. To validate this concept, an experiment was performed to evaluate the effects of a complete range of red:blue ratios on 7 plant species: *Arabidopsis thaliana, Brachypodium distachyon, Euphorbia peplus, Ocimum basilicum, Oryza sativa, Solanum lycopersicum*, and *Setaria viridis*. Plants were exposed during 30 days to the light gradient and showed significant, but species-dependent, responses in terms of dimension, shape, and color. A time-series analysis of phenotypic descriptors highlighted growth changes but also transient responses of plant shapes to the red:blue ratio.

**Conclusion:**

This approach, which generated a large reusable dataset, can be adapted for addressing specific needs in crop production or fundamental questions in photobiology.

## Introduction

New urban agriculture business models are emerging as market demand for local production of high-quality fruits and vegetables is increasing [1]. This, in turn, is stimulating the development of techniques used in controlled environment agriculture (CEA), offering unique opportunities for year-round production, independently of season, weather, soil conditions, or climate change, as well as reduced resource use and lower production costs [2, 3].

The economic feasibility of CEA owes a lot to the development of light-emitting diode (LED) technology, which progressively replaces traditional artificial lighting sources. Indeed, LED lighting fixtures show a great potential for energy saving compared to former technologies (e.g., high-pressure sodium lamps) [4, 5]. In addition, they provide control over spectral composition, flexible fixture format, durability, long operating lifetime, relatively cool emitting surfaces, and a photon output that varies linearly with electrical input current [6]. These attributes can greatly facilitate the application of photobiology at all stages of crop production, from propagation to postharvest quality control. Besides providing energy for photosynthesis, light indeed plays a key role in many plant responses that depend on its duration, intensity, and spectrum, which are perceived by a battery of photoreceptors [7–9]. It can thus be expected that LED will revolutionize indoor crop production [5], all the more as the technology is still improving in efficiency while capital costs keep decreasing [10].

Interestingly, CEA has its own breeding targets. Indeed, in addition to indoor-specific constraints (e.g., small size and short cycle), the desired plant's response to the environment resides in phenotypic plasticity rather than resilience to stress conditions [11]. For example, different light qualities could be used to grow the same lettuce genotype for different products such as green versus red salads [12]; therefore genotypes that show such plasticity are desirable.

In the context of these fast technological developments, screening for CEA-specific breeding targets and optimizing environmental conditions for new business models are key steps. Meeting these needs efficiently requires high-throughput approaches, such as those used for plant phenomics [13]. Phenomics is a relatively recent research field, initially triggered by the huge demands for phenotyping capacity in functional genomics studies [14]. It has been focused primarily on model plants such as *Arabidopsis thaliana*, as well as large-scale crops such as cereals and other major productions. Phenomics relies heavily on imaging technologies that are non-destructive and allow the quantification of complex structures in a fast and highly repeatable way. Correlation between image-based descriptors and ground-truth data obtained by direct measurements has been demonstrated multiple times in different model systems. For example, (i) projected leaf area or height has been shown to correlate well with direct measurements of plant dimensions and biomass in wheat [15], *Arabidopsis* [16, 17], or tomato [18]; (ii) geometric descriptors have been used to objectivize shape variations between *Arabidopsis* genotypes [19, 20]; and (iii) color indices based on simple red green blue (RGB) images have proven useful for discriminating differences in leaf chlorophyll content, e.g., in soybean or corn canopies [21, 22]. Furthermore, several studies demonstrated that descriptors extracted from high-throughput imaging, such as plant area or volume, can be used as non-destructive estimators of shoot biomass [15, 17, 23]. The requirement for high-throughput phenotyping increases as the plant research community addresses the future challenges that agriculture will face with climate change [24]. Obviously, the same technological advances in sensors, imaging, automation, and data processing that benefit functional genomics can be used to evaluate plant phenotypes under indoor cultivation contexts, as well as to identify either opti-mum conditions for available genotypes or fitter genotypes for indoor conditions.

A timely research investment for CEA development is thus to use plant phenomics to explore the many new avenues, constraints, and needs that currently emerge from the rapid worldwide adoption of LED technology. Previous studies aiming at evaluating the effects of light quality on plant production mostly compared limited numbers of discrete conditions (e.g., different ratios of red:blue, red:far-red, % UV) within very specific combinations of target species/genotypes, environments, traits of interest, and phenotyping approaches [6]. As light sources and growing set-ups widely differ across laboratories, customizing the lighting conditions for each economically important plant remains complex, and knowledge gaps still limit the productivity of CEA [13]. Therefore, a more comprehensive method to characterize plant phenotypic responses to light quality is desirable and would also provide a boost to basic photobiology research in model systems.

In this article, we examine the methodological advances provided by light quality gradients in terms of phenotyping speed, logistics, and information content, and whether this would facili-tate studies of light quality responses. To our knowledge, light gradients have seldom been studied as such, except in agroecology contexts such as forestry, where irradiance is the main variable factor [25–27]. Therefore, light quality gradients represent a new experimental approach offering several potential advantages: (i) a wide range of spectral ratios can be tested in 1 cycle, while all other parameters remain constant; (ii) the continuous variation in light quality offers the possibility of detecting thresholds, peaks, and troughs in the plant response; (iii) regressions can be used to estimate correlation, effect size, and significance in an easy and straightforward way; and (iv) when combined with non-destructive phenotyping methods such as time-series imaging, they provide detailed information on the plasticity of various target traits.

A multi-species experiment was designed to test a gradient of red and blue lights because these colors have been the focus of many publications in the horticultural domain [6, 28, 29]. Smart LED luminaries were used to create a continuous range of red:blue ratios under otherwise constant conditions, and an imaging platform was used to measure basic phenotypic traits related to growth, morphology, and pigmentation of the plants (plant dimensions, shape factors, color indices). Among the numerous options for digital imaging set-ups that have been developed for a variety of applications and scientific questions [30], a simple low-cost design was used, based on off-the-shelf electromechanics, RGB cameras, and open-source image acquisition and analysis software. Depending on the purpose, such “maker-made” phenotyping stations can provide sufficient image quality and throughput as shown in a growing number of publications [31–33].

Seven plant species were selected, on the basis of their scientific and economic importance, as well as botanical and architectural diversity. Four dicot species were used: *A. thaliana* (Brassicaceae), an obvious choice owing to its importance in academic research and the wealth of genomic and phenomic knowledge; *Solanum lycopersicum* (Solanaceae) and *Ocimum basilicum* (Lamiaceae), 2 interesting models for horticultural applications; and *Euphorbia peplus* (Euphorbiaceae), a wild species studied for its medicinal properties. Three monocot species (Poaceae) were also grown: 1 temperate species, *Brachypodium distachyon*; 1 tropical crop, *Oryza sativa*; and 1 C4 wild species, *Setaria viridis*.

## Results and Discussion

### Data description

**Figure 1:**
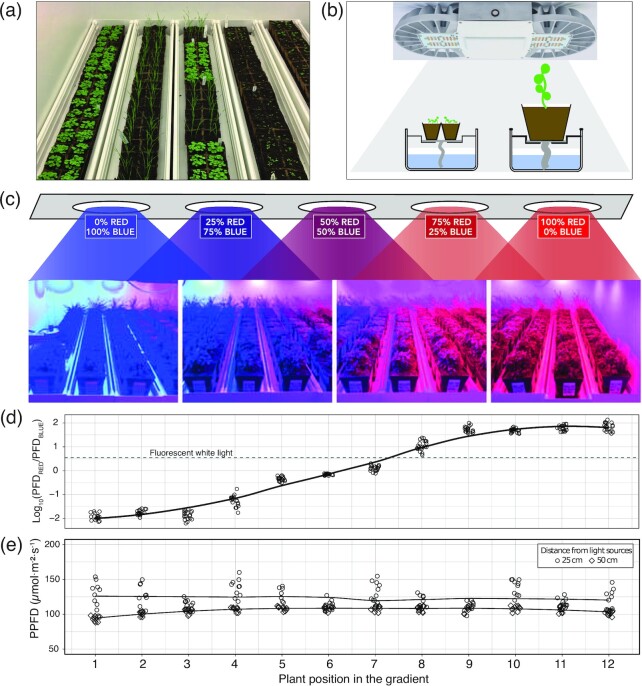
Cultivation set-up under red:blue light gradient. (a) 30-day-old plantlets at the end of pre-cultivation period. (b) Cultivation system before (small pots) and after (large pots) transfer under the red:blue light gradient. (c) Red:blue gradient setup. Arrangement and setting of the 5 clusters of Lumiatec PHS::16 luminaries in the phytotronic cabinet. (d) Red:blue ratio measured at each plant position; PFD: photon flux density. (e) Total light irradiance measured across the gradient; PPFD: photosynthetic PFD.

Plants of 7 different species were grown 30 days under white light, then transferred under a gradient of red to blue LED lights for another 30 days, and finally returned to white light (Fig. 1). Phenotypic data were collected twice a week from side- and top-view images of individual pots. Image processing delivered 3 types of phenotypic descriptors: (i) simple dimensions (e.g., height, width, projected area, fitted ellipse), (ii) shape factors derived from simple dimensions (e.g., roundness, solidity, circularity), and (iii) color mean density values (red, green, blue, hue, saturation, brightness) and their respective standard deviations. A detailed explanation of the phenotypic descriptors is provided in Table 1.

**Table 1:** Plant dimension, shape, and color parameters measured by imaging: definition, calculation, and units

Label	Definition	Formula	Unit or scale
**Dimensions**			
Side-view HeightMax	Maximum height out of 6 side-view images during 180° rotation		mm
Side-view WidthMax	Maximum width out of 6 side-view images during 180° rotation		mm
Side-view AreaMean	Mean projected area out of 6 side-view images during 180° rotation		mm²
Top-view Area	Projected area out of 1 top view image		mm²
Top-view MeanFeret	Mean of maximum and minimum distances between 2 points along the selection boundary		mm
Voxel	Plant volume estimate combining side- and top-view area of the plant	sqroot [max(side-view area) * min(side-view area) * top-view area]	mm³
**Shape factors**			
Side- and Top-view Roundness	Degree of similarity to a circle derived from the fitted ellipse axes	minor axis/major axis (of the fitted ellipse)	Scale 0–1
Side- and Top-view Solidity	Overall concavity derived from area and convex-hull measurements	area/convex-hull area	Scale 0–1
Side- and Top-view Convexity	Edge “roughness” derived from convex hull and perimeter measurements	convex-hull perimeter/perimeter	Scale 0–1
Side- and Top-view Circularity	Ratio of the area of the shape to the area of a circle having the same perimeter (a.k.a.“isoperimetric quotient”)	4π * area/perimeter²	Scale 0–1
Side- and Top-view Compactness	Degree of compacity derived from the ratio of the diameter of a circle with the same area to the major axis of the fitted ellipse	sqroot[(4/π) * area]/major ellipse axis	Scale 0–1
**Color indices**			
Side- and Top-view HueMean	Mean hue component of the plant's color after transformation of the RGB image into HSB model		Scale 0–255
Side- and Top-view HueCv	CV of the plant's pixels' hue	stdev(hue)/avg(hue) ^*^100	%
Side- and Top-view SaturationMean	Mean saturation component of the plant's color after transformation of the RGB image into HSB model		Scale 0–255
Side- and Top-view BrightnessMean	Mean brightness component of the plant's color after transformation of the RGB image into HSB model		Scale 0–255
Side- and Top-view RedMean	Mean red component of the plant's color in the RGB model		Scale 0–255
Side- and Top-view GreenMean	Mean green component of the plant's color in the RGB model		Scale 0–255
Side- and Top-view BlueMean	Mean blue component of the plant's color in the RGB model		Scale 0–255
Side- and Top-view Density	Integrated density: the sum of the grey values of the pixels in the image or selection	area * mean grey value	
Top-view GLI	Green leaf index: vegetation index for use with a digital RGB camera	(2 * green - red - blue)/(2 * green + red + blue)	
Top-view TGI	Triangular greenness index: approximate area of a triangle bounding a leaf reflectance spectrum, where the vertices are in the red, green, and blue wavelengths	[(670 – 480) * (red - green) - (670 – 550) * (red - blue)] / −200	
Top-view Chl_predicted	Predicted leaf chlorophyll content derived from multiple linear regression using red, green, and blue components of the plant color in the RGB model	440 + blue * 7.266 + red * 10.873 + green * −15.545	µmol m^–^²

CV: coefficient of variation; GLI: green leaf index; HSB: hue saturation brightness; TGI: triangular greenness index.

**Figure 2:**
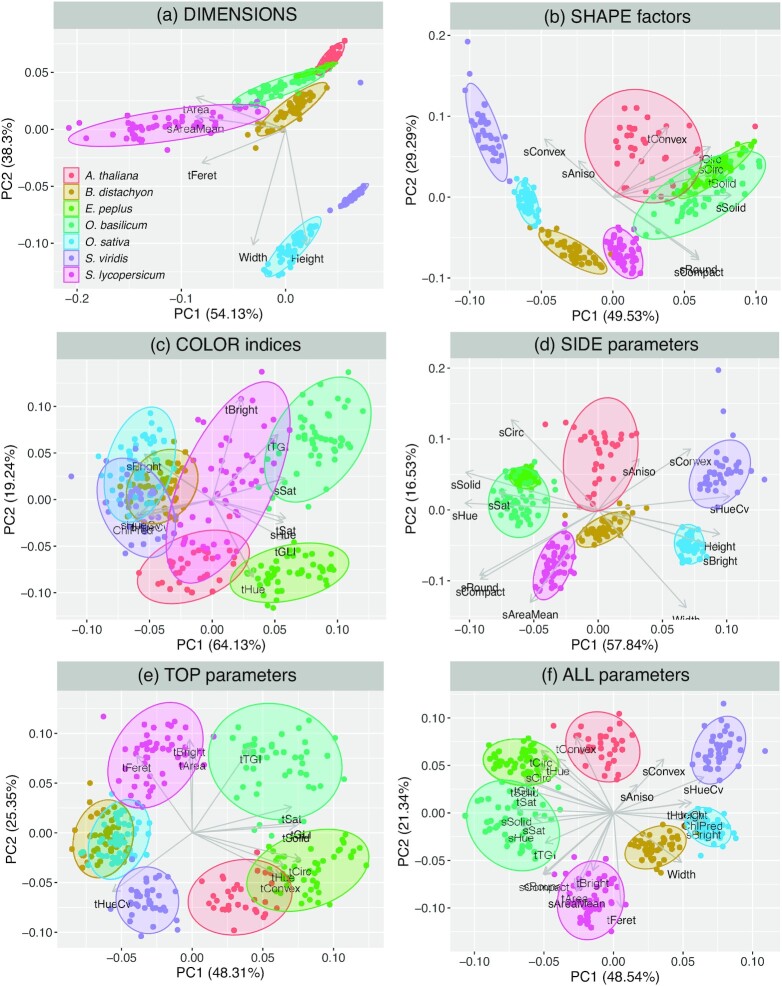
Principal component analysis discrimination of 7 species based on various selections of phenotypic descriptors. Species color codes in panel (a). Imaging data collected over 3 timepoints between 21 and 29 days after transfer under red:blue gradient were used.

The dataset was first used to evaluate the potential of the imaging platform to discriminate diverse plant species and morphologies, from narrow-leaf monocots (*B. distachyon, O. sativa, S. viridis*) to large-leaf caulescent tomato (*S. lycopersicum*) or multi-plant bushes (*E. peplus, O. basilicum*). Figure 2 shows how species discrimination by principal component analysis (PCA) performed, based on different combinations of the 3 types of phenotypic descriptors (dimensions, shape factors, color indices) and the 2 camera views (side- and top-views). As expected, the different species were best discriminated based on the full set of descriptors, all other combinations yielding only partial separations, especially for the 3 monocots. A main limitation was also found with *A. thaliana*, whose basal rosette of flat leaves could only be characterized consistently from the top-view images.

To evaluate the effect of the red to blue LED gradient for each plant species, a linear regression was calculated for each phenotypic descriptor against the log-transformed red:blue ratios measured at each plant location. Besides recording Pearson *R* and *P*-value, the slope and intercept of the regression were used to estimate descriptor values at both the minimal and the maximal red:blue ratios. The difference between these values was defined as the “effect size” of the gradient, which is expressed as the percentage difference across the red:blue gradient. The regression graphs can be generated using the R scripts provided in the “Source Code” section. These calculations were performed at each phenotyping time point in order to evaluate the variation of the red:blue ratio effects during and after the gradient treatment.

### Differential growth, shape, and color under variable red:blue ratio

**Figure 3:**
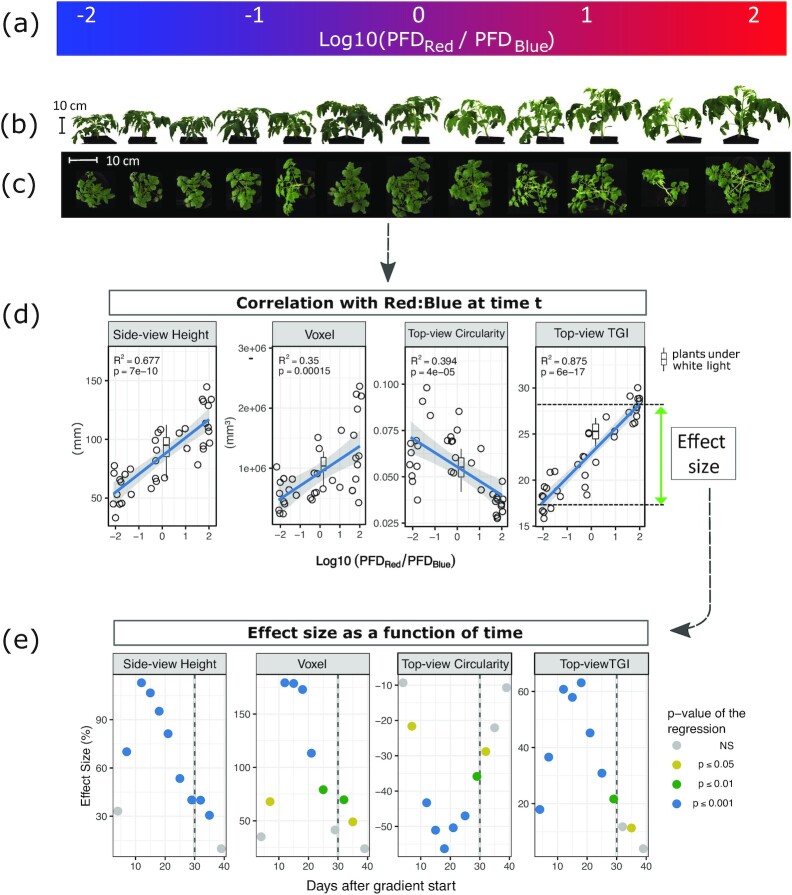
Example of plant phenotypes in *S. lycopersicum* under the red:blue gradient. (a) Light gradient. PFD: photon flux density. (b) Side-view and (c) top-view images of a row of tomato plants 21 days after transfer to the gradient conditions. (d) Use of linear regression to estimate correlation (*R*^2^), significance (*P*-value), and “effect size” (% difference across the gradient) for some descriptors. The box-and-whiskers represent the descriptor variation observed in plants grown under white light. The horizontal line is the median and boxes are bound by 25th and 75th percentiles with whiskers extending 1.5 interquartile range (IQR). (e) Variation of the gradient “effect size” as a function of time for the same descriptors as in (d). NS: nonsignificant.

Figure 3 shows the kinds of images and data that were obtained for tomato (*S. lycopersicum*), as an example. Plants were visually taller, wider, and bulkier as the red:blue ratio increased (Fig. 3b and c). Image-based phenotypic descriptors allowed these effects on plant height and volume to be quantified (estimated by Voxel descriptor), and revealed more subtle changes, such as a decrease in circularity, a shape factor that quantifies area:perimeter variation (Fig. 3d). This was likely due to the elongation of stems and petioles, which increased the convexities in the plant contours under high red:blue ratio. The triangular greenness index (TGI) calculated from RGB density values also increased, indicating higher reflectance in the green broadband (Fig. 3d). Because, as expected from the literature [22, 34], TGI was negatively correlated with chlorophyll content estimates (see Supplementary Fig. S3), this color change suggested a decrease in leaf chlorophyll content with higher red:blue ratios. This combination of phenotypes is consistent with previous studies showing that blue wavelengths reduce stem elongation and increase chlorophyll concentration in *S. lycopersicum* [35, 36]. Interestingly, repeating the phenotyping procedure during and after the red:blue treatment revealed that the effect size of the gradient changed over time. It was strongest 2 weeks after the start of the treatment for a number of descriptors (Fig. 3e) but diminished markedly afterwards, suggesting a possible acclimation process.

**Figure 4:**
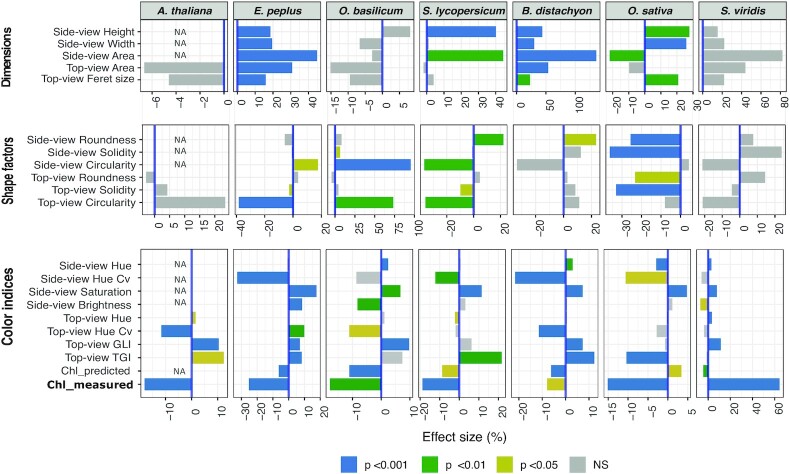
Effect size of the red:blue gradient on different phenotypic descriptors estimated at the last imaging point before re-transfer to white light (29 days after the start of the gradient). The significance categories are based on the *P*-value of the computed *R*^2^. Side-view data for *A. thaliana* are not shown (NA). Note that “Chl-measured” is not an imaging-based index, but an estimate of leaf chlorophyll content obtained with a handheld probe (Apogee MC-100). NS: nonsignificant.

Similar analyses were performed for the other 6 species. Figure 4 shows the calculated “effect sizes” of the red:blue gradient at the end of the light treatment for 20 phenotypic descriptors that showed a highly significant correlation (*P* < 0.01) with the red:blue ratio in ≥1 species. Although the effects on height and color described above for *S. lycopersicum* were mostly consistent across species, the pattern and amplitude of the effects on the full array of phenotypic descriptors appeared highly species-specific. For example, in *S. lycopersicum*, effects on dimension descriptors were observed in side-view images only, while in *E. peplus, B. distachyon*, and *O. sativa*, top-view dimensions were also affected, and in *A. thaliana, O. basilicum*, or *S. viridis*, no significant effects on any dimension descriptors were observed. An increase in plant height with the red:blue ratio is reported in the horticultural literature involving phylogenetically distant eudicots such as cabbage [37], artichoke [38], cucumber [39], tomato [35, 36], or lettuce [40]. In *O. basilicum*, however, which is probably one of the most studied species under indoor conditions including LED lights, previous reports showed conflicting results. For example, blue light was reported to affect stem elongation and leaf expansion either positively [41, 42] or negatively [43]. These discrepancies demonstrate the difficulty of comparing phenotypic studies performed in different laboratories where cultivation set-ups vary, and strengthen the interest in using an LED color gradient to change light quality with all other environmental parameters being constant.

**Figure 5:**
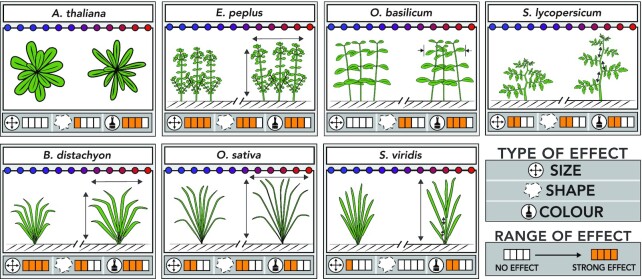
Schematic representation of the phenotypic variations caused by a red:blue light gradient in 7 plant species. Effects observed 4 weeks after the start of the light gradient.

In terms of shape descriptors, all species exhibited different patterns of responses to the red:blue ratio, which was expected because of their different architectures. Nevertheless, the combination of descriptors made it possible to capture how light quality affected the general appearance of the plants, as sketched in Fig. 5. Interestingly, some of these effects were known in the literature, which supports the suitability of the imaging pipeline developed here.

For example in *A. thaliana*, higher red:blue ratios induced curling of the leaves that were also slanted downwards [44], and this was captured here by a decrease in top-view circularity, as leaves were seemingly narrower when seen from the top (Figs   4 and 5). A similar phenotype, known as part of the “red-light syndrome,” has been reported in other species, including tomato [45] and cucumber [46].

In *B. distachyon*, which is the species whose shape and size were the most affected by light quality (Figs 4 and 5), side- and top-view area, height, and width increased with the red:blue ratios, which could be explained by a reduction of the foliage by blue light, as already reported for wheat [47], and/or an increase of branching (tillering) by red light, as reported in red:far red experiments [48, 49]. In *O. sativa*, increased red:blue ratios altered the plant shape by enhancing the erectness of leaves and causing plant tightening, as indicated by changes in both side- and top-view roundness and solidity descriptors (Figs 4 and 5). Interestingly, erect leaves were previously shown to improve photosynthesis and yield in rice by reducing leaf shading in dense plantations [50]. This phenotype is regulated by environmental and hormonal factors, among which brassinosteroids exert a prominent role. The effects of light quality observed here could thus act upstream of these hormones, as suggested by Asahina et al. [51].

The color indices green leaf index (GLI), TGI, or predicted chlorophyll content (Chl-predicted) all pointed towards a decrease in chlorophyll content with higher red:blue ratio, which is in line with reported effects of red and blue lights in various species such as lettuce [40], cabbage [37], tomato [36], cucumber, pepper, or radish [35]. Also, the expected negative correlation between TGI and chlorophyll content estimates [22, 34] was found in most species (Supplementary Fig. S3). There were 2 exceptions, however: (i) the correlation between TGI and chlorophyll estimates was reversed in *O. sativa* (Supplementary Fig. S3), possibly as a consequence of leaf inclination and reflectance changes with light quality; and (ii) there was no correlation between TGI and chlorophyll estimates in *S. viridis*, and the effect of the red:blue ratio on chlorophyll estimates was opposite to what was observed in the other species (Fig. 4). It is tempting to speculate that this peculiar behavior of *S. viridis* is linked to its C4 metabolism, but information on this topic is scarcely available in the literature. In one report on maize, though, it was shown that blue light represses the accumulation of chlorophylls, compared to red light [52]. Concerning the lack of correlation between TGI and chlorophyll estimates, one explanation might be that *S. viridis* plants started flowering during the gradient treatment, and TGI may have been biased by the presence of paler green panicles, independently of the variations in leaf chlorophyll content. This is a good reminder that chlorophyll content is not always the main explanatory variable in a color index. Indeed, although RGB reflectance was shown to be a good chlorophyll proxy in different species [21, 53, 54], it lacks specificity and is sensitive to other pigments as well as to leaf texture and/or inclination.

### Patterns of change over time

**Figure 6:**
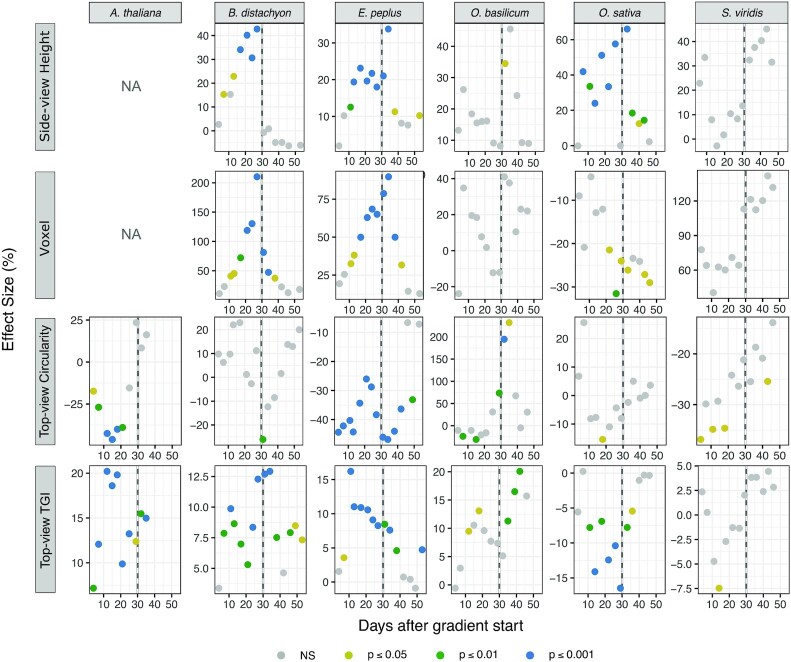
Time-course variation of the “effect size” of the red:blue gradient for 4 phenotypic descriptors in 6 species. Vertical dotted line: end of the red:blue gradient treatment and return to white light. The significance categories are based on the *P*-value of the computed *R*^2^. Side-view data for *A. thaliana* are not shown (NA). NS: nonsignificant.

An undeniable advantage of image-based phenotyping is that it allows repeated measurements and thus provides a dynamic output. It was important in this study because the effect size of the red:blue gradient was found to change over time, but in different ways in the different species (Fig. 6). As mentioned above, the effect of the red:blue gradient was transient in tomato, being strongest 2 weeks after the start of the treatment for a number of descriptors (Fig. 3d). By contrast in *B. distachyon, O. sativa*, and *E. peplus*, the effect size increased with time and then diminished slowly after return to white light (Fig. 6). In *O. basilicum*, no significant effect was observed during exposure to the gradient, but the transfer back to white light caused sudden and transient changes in parameters such as height, circularity, and TGI, indicating re-adjustment of plants to the light quality fluctuation.

Plant acclimation to light fluctuations is subject to immense interest but is mostly focused on irradiance rather than spectrum fluctuations [55, 56]. In nature however, intensity and quality fluctuations are often concomitant. For example, plants undergoing shading in a canopy experience both a decrease in intensity and a shift towards greener light with lower red:far red ratio. In that respect, Wagner et al. [57] showed that the long-term response to fluctuating light quality is an important and distinct light acclimation mechanism that supports survival of *A. thaliana* under low light conditions, and hence integrating time into the understanding of plant responses to light quality deserves more attention. An “end of treatment” phenotyping would undoubtedly miss important data.

## Conclusion

It is clear from this study that the effects of light quality on plant phenotypes are strongly species-dependent, so no predictive clues can be generalized. Experimentation is thus absolutely required before any application of LED technology in CEA or other research contexts. In that respect, this study demonstrates that, compared to discrete conditions, the use of a light gradient allows subtle phenotypic effects to be captured while avoiding the interference of other environmental variations, which still hampers comparisons of data acquired in different plant growth facilities. The analysis of the dataset provided here also shows that high-throughput phenotyping is required to capture the complexity of plant plasticity and that a time course is needed to measure possible transient effects.

Additional features could improve the platform described here. For instance, throughput could be increased by adding automated steps (plant conveyors or moving top-view cameras on gantry) that were not included in our maker-made platform but are quite common for higher capacity facilities [58–60]. The accuracy, relevance, and depth of imaging could be improved by using new technologies such as spectral, tridimensional, thermal, or fluorescence cameras, depending on the desired application and/or traits of interest. In particular, this would address the pertinence of the RGB color indices and the biases caused by plant shapes and leaf inclination, which were also reported in studies on spectral imaging [61]. Image analysis could be accomplished with other available software, some of which offer more specialized functionalities than the free and popular generalist package, ImageJ, which was used here. The online resource [62] curates currently available tools for morphological plant image analysis [63]. More elaborate data processing could also be explored beyond linear regression, while machine learning approaches could facilitate the interpretation of the complex set of parameters generated by imaging. For example, classification techniques would allow plants to be categorized according to predefined criteria and provide the user of the dataset with a more holistic understanding of the plant phenotype.

All these perspectives further broaden the potential advantages of combining LED gradients with imaging-based plant phenotyping for both environmental optimization and genotypic selection of CEA targets. The methodology can be adapted to multiple use-cases by changing the LED wavelengths, the gradient configurations, and the timing of the light treatments.

## Methods

### Plant materials

*Arabidopsis thaliana* Col-0 seeds were obtained from a public seedbank (NASC, Nottingham, UK) and *Brachypodium distachyon* Bd21-3 seeds from Prof. R. Amasino (University of Wisconsin, Madison, WI, USA). Seeds of *Euphorbia peplus* were obtained from fairdinkumseeds.com (Gin Gin, Queensland, Australia). Seeds of *Setaria viridis* A10.1 were obtained from USDA Iowa State University Agricultural Research Service (Ames, IA, USA). Seeds of *Solanum lycopersicum* cv Ailsa Craig were obtained from TGRC (Davis, CA, USA). *Ocimum basilicum* cv Genovese seeds were obtained from Le Jardin de Bellecourt (Bellecourt, Belgium). Seeds of *Oryza sativa cv*. Nipponbare were obtained from IRRI (Los Baños, Laguna, Philippines). All materials were obtained and used within the Rights and Obligations of the Recipient as specified by the International Treaty on Plant Genetic Resources for Food and Agriculture adopted by the FAO Conference on 3 November 2001 and entered into force on 29 June 2004.

### Growth conditions

Seeds were sown in 4.5-cm fiber pots (Jiffypots®, Jiffy, Zwijndrecht, Netherlands) filled with a 4:1 mix of leaf mold and baked clay granules. The fiber pots were placed on 120 × 18 × 14 cm cultivation gutters (Goponic, Nouméa, France) and irrigated by capillarity through a wet cultivation felt mat (Feutriplanta®, Jardirama, Warsage, Belgium) and wicks dipping in the water (Fig. 1a and b). The gutters were placed for 30 days in a Conviron PGV36 phytotronic cabinet (Conviron, Winnipeg, MB, Canada) at 21°C day/night, 70% relative humidity under 12-h photoperiod provided by Sylvania Luxline Plus T5 FHO 54W tubes (Osram-Sylvania, Wilmington, MA, USA) delivering 4,000K white light (see spectral distribution data in Supplementary Fig. S2). Photosynthetic photon flux density (PPFD) (400–700 nm) was adjusted to ±130–150 µmol m^–2^ s^–1^, as measured using a HiPoint HR-550 spectrophotometer (Taiwan Hipoint Corp., Kaohsiung, Taiwan). After 4 weeks, Jiffypots were transplanted into 12-cm square plastic pots filled with the same substrate, supplemented with 6 g L^–1^ of slow release fertilizer (Osmocote Exact Standard 5–6 M, ICL Specialty Fertilizers). Only 1 plant per pot was kept, except for *E. peplus* (6 plants/pot) and *O. basilicum* (up to 9 plants/pot) to account for their usual mode of cultivation in bushes. The pots were fitted at the bottom with a 2 × 10 cm felt wick for capillarity irrigation and randomly placed on the deck of the cultivation gutters. The gutters were then placed in the same environmental conditions as previously, except for the lighting, which was provided either by white fluorescent tubes as before or by adjustable Lumiatec PHS::16 luminaries providing a range of red:blue light ratios (described below and Fig. 1c). The carbon dioxide concentration was ambient and remained within 390–410 ppm throughout the experiment (measurements performed with IRGA analyzer WMA-5 PP Systems, Amesbury, MA, USA).

### Red-blue light gradient

The phytotronic cabinets were equipped with 15 Lumiatec PHS::16 (300W) luminaries (GDTech, Alleur, Belgium) each. These luminaries are controllable over 16 channels and fitted with PCB-LEDs offering 2 × 6 Blue LED 455 nm, 6 × 6 White LED 4,000K, 1 × 6 Green LED 520 nm, 1 × 6 Yellow LED 593 nm, 2 × 6 Red LED 635 nm, 2 × 6 Hi-Red LED 660 nm, 1 × 6 Far-Red LED 730 nm, and 1 × 6 UV LED 280 nm. The 15 luminaries were regularly distributed at a distance of 0.5 m (Fig. 1c) and were controlled per cluster of 3 using the Lumiatec control interface. The blue and hi-red channels (see spectral distribution data in Supplementary Fig. S2) were adjusted as shown in Fig. 1c to create a gradient of red:blue ratio (Fig. 1d). The light spectrum and intensity above each plant were monitored using the same HiPoint HR-550 spectrophotometer as before. PPFD under the gradient conditions was 100–150 µmol m^–2^ s^–1^ (Fig. 1e). The other parameters used during the gradient treatment (air temperature, relative humidity, photoperiod) were the same as described in the “Growth conditions” section.

#### Layout under red:blue gradient

Each room (3 m²) allowed 12 gutters of 10 pots (Fig. 1c). The placement of the plants was organized in rows and columns so that each pot could be registered by room:row:column coordinates and labeled with a unique quick response identifier (QR-code). The gutters corresponded to the columns, and there were 36 pots per species in 3 contiguous rows of 12 pots, except for *Arabidopsis*, which had 4 rows, so 48 pots. No guard rows were used. At the beginning of the LED gradient treatment, a randomization step was performed for each species within their block, after which the pots were kept at the same position until the end of the treatment. A detailed representation of the layout is provided in Supplementary Fig. S1. On Day 60, the plants were all transferred back to white light conditions, and the experiment was stopped 4 weeks later.

### Imaging hardware

Plants grown in individual pots were placed on a rotating platform and photographed laterally (6 side-view images during a 180° rotation) or from the ceiling (1 top-view image). The imaging set-up was built with aluminium profiles supporting white diffusive polyvinyl chloride walls. The cabinet was illuminated by 25 × 25 cm white light LED panels (Araponics, Liège, Belgium). Lighting was optimized for taking pictures with a diffusive back-lit white background for side-view images and a black cloth background for top-view images. A step-motor platform was used to rotate the pot while 2 color (RGB) 12 Mpx cameras (Dalsa Genie-nano 4040, Dalsa, Waterloo, ON, Canada) acquired plant images from side and top view and 1 color HD webcam (Logitech, Lausanne, Switzerland) read QR-coded labels on the pot. Spatial specifications allowing reproduction of this set-up are provided in Supplementary Fig. S4. The Genie-Nano cameras were fitted with high-resolution 25-mm focal length Tamron M111FM25 lenses, which enabled imaging of plants ≤150 cm high and 100 cm wide with an estimated smallest detail size of ±0.5 mm at a working distance of 200 cm, based on sensor dimensions (14.2 × 10.4 mm, 4,112 × 3,008 pixels) and lens optical resolution (3.1 µm “pixel pitch”). Diaphragm closure of the lenses was set to F8.0, exposure time to 0.2 msec, and gain to 6. The cameras and the stepper-motor were controlled through dedicated software written in Python and running on a Linux computer to synchronize plant identification, rotation, and image acquisition. The adjustment of basic camera settings (e.g., shutter speed, gain, output format) used libraries from OpenCV (OpenCV, RRID:SCR_015526) [64] and Aravis [65], while rotation functionalities (i.e., speed, number, and time of acquisitions after QR-code detection) were programmed by us. Typically, 6 side-view images and 1 top-view image were acquired during a 180° rotation in 4 seconds (45° per second). The pots were manu-ally loaded on the rotating platform through a sliding door. After rotation was initiated, the imaging cycle started when the QR-code was read by the webcam, and each image acquired by the Genie-Nano cameras was saved under a unique identifier (UID). The complete imaging cycle was ∼10 seconds per pot.

### Image processing and generation of phenotypic descriptors

An automated script was developed using the macro language of the ImageJ open source package (Fiji distribution) [66] to extract plant phenotypic descriptors from each image. The successive steps were (i) reading the raw image in Bayer format; (ii) getting metadata, e.g., date, pot UID, camera view, frame No.; (iii) white balance and spatial calibration based on a reference color chart; (iv) segmentation of the plant from background using grey-scale or color thresholding; (v) measurement of plant dimensions and shape factors; (vi) extraction of color components in either RGB or HSB (hue saturation brightness) color space; (vii) exporting raw data in text format (.csv).

R version 3.6.1 for macOSX [67] running under Rstudio version 1.3.1093 (Rstudio, Boston, MA, USA) was used to (i) compute additional shape factors as ratios from existing measurements, such as voxel, compactness, anisotropy; (ii) compute color indices such as GLI and TGI; (iii) generate a chlorophyll content prediction based on RGB values; (iv) generate scatter plots to visually check for abnormal measurements due to, e.g., corrupted images, before further statistical use; (v) aggregate the multiple camera measurements per pot (e.g., the side-view camera generated 6 images from which the mean, maximum, minimum, and median values were computed); (vi) merge imaging data with plant metadata (species, spatial location, intensity and quality of light at plant location, estimated chlorophyll content). A more detailed description of the image processing is found in Supplementary Table S1.

### Chlorophyll content estimate

The leaf chlorophyll content was estimated with a hand-held probe measuring the transmittance ratio of cell walls at 931 nm versus chlorophyll at 653 nm (Apogee MC-100, Apogee Instruments, Logan, UT, USA). These measurements were performed once, at the end of the red:blue gradient treatment. Species-specific calibration models provided with the instrument were used for tomato and rice, whereas a generic model, averaged from multiple species (described in [68]) was used for the other species. At least 6 measurements were made on a minimum of 3 different mature leaves per pot. The measurements were averaged per pot.

## Supplementary Material

giab101_GIGA-D-21-00184_Original_Submission

giab101_GIGA-D-21-00184_Revision_1

giab101_Response_to_Reviewer_Comments_Original_Submission

giab101_Reviewer_1_Report_Original_Submission Yujin Park -- 8/10/2021 Reviewed

giab101_Reviewer_2_Report_Original_Submission Filippos Bantis -- 8/16/2021 Reviewed

giab101_Reviewer_2_Report_Revision_1 Filippos Bantis -- 11/19/2021 Reviewed

giab101_Supplemental_Files

## Data Availability

Raw images, image analysis script, and raw data file are available at zenodo.org [69].

## Additional Files

**Figure S1:** Experimental design and layout during the gradient treatment. Four phytotronic cabinets were used. Three of them (G4, G5, G9) were equipped with Lumiatec PHS::16 luminaries and 1 (G8) was equipped with regular fluorescent tubes (white light). Three successive sowings were performed at 2 weeks interval in chamber G8. Each sowing consisted of 2 species and *A. thaliana*. After 30 days in G8, 36 plants of each species (48 for *A. thaliana*) were transferred to the respective Lumiatec PHS::16-equipped phytotronic cabinet for the gradient treatment. Twelve plants of each species per sowing were kept under white light in G8 until the end of the experiment. Each plant was tagged with a unique identifier. Note that *A. thaliana* plants in chambers G4 and G5 were sown for molecular analyses that are not reported in this article.

**Figure S2:** Spectral distribution of the red and blue LED of the Lumiatec PHS::16 luminaries and the white fluorescent lights used in the experiment.

**Figure S3:** Correlation between leaf chlorophyll content, as estimated manually with an Apogee MC-100 chlorophyll meter, and the triangular greenness index (TGI) computed from RGB images. Measurements of both TGI and chlorophyll content in this figure were performed 29 days after the start of the gradient treatment.

**Figure S4:** Blueprint of the imaging cabinet.

**Table S1:** Steps in the image processing to generate plant shape and color proxies.

## Source Code

R script for statistical analysis, subsequent processed data files, and plot files are available at codeocean.com [70].

## Abbreviations

CEA: controlled environment agriculture; GLI: green leaf index; HSB: hue saturation brightness; LED: light-emitting diode; PCA: principal component analysis; PCB: printed circuit board; PFD: photon flux density; PPFD: photosynthetic photon flux density; QR-code: Quick Response code; RGB: red green blue; TGI: triangular greenness index.
