# Supplementary material for: LED color gradient as a new screening tool for rapid phenotyping of plant responses to light quality
Source: Gigascience. 2022 Jan 27;11:giab101. doi: 10.1093/gigascience/giab101 (PMC8848316; doi:10.1093/gigascience/giab101)

GigaScience

Led Color Gradient As A New Screening Tool For Rapid Phenotyping Of Plant Responses To Light Quality

--Manuscript Draft--

**Manuscript Number:**	GIGA-D-21-00184
**Full Title:**	Led Color Gradient As A New Screening Tool For Rapid Phenotyping Of Plant Responses To Light Quality
**Article Type:**	Research
**Funding Information:**	european funds for regional development (feder) 2014-2020 — Not applicable<br>service public de wallonie (spw) - wagralim competitiveness cluster — Not applicable<br>fonds de la recherche scientifique - fnrs (FC87200) — Dr Frédéric Bouché<br>fonds pour la formation à la recherche dans l'industrie et dans l'agriculture (FC21283) — Mr Samuel Huerga-Fernández
**Abstract:**	Background<br><br>The increasing demand for local food production is fueling high interest in the development of controlled environment agriculture (CEA). In particular, LED technology brings energy-saving advantages together with the possibility to manipulate plant phenotypes through light quality control. However, optimizing light quality is required for each cultivated plant and specific purpose.<br><br>Findings<br><br>In this paper, we show that the combination of LED gradient setups with imaging-based non-destructive plant phenotyping constitutes an interesting new screening tool with the potential to improve speed, logistics, and information output. To validate this concept, an experiment was performed to evaluate the effects of a complete range of Red:Blue ratios on seven plant species: Arabidopsis thaliana , Brachypodium distachyon , Euphorbia peplus , Ocimum basilicum , Oryza sativa , Solanum lycopersicum , and Setaria viridis . Plants were exposed during four weeks to the light gradient and showed significant, but species-dependent, responses in terms of dimension, shape, and color. A time series analysis of phenotypic descriptors highlighted growth changes but also transient responses of plant shapes to the Red:Blue ratio.<br><br>Conclusion<br><br>This approach, which generated a large reusable dataset, can be adapted for addressing specific needs in crop production or fundamental questions in photobiology.
**Corresponding Author:**	Claire Périlleux, PhD<br>University of Liège<br>Liège, BELGIUM
**Corresponding Author Secondary Information:**	
**Corresponding Author's Institution:**	University of Liège
**Corresponding Author's Secondary Institution:**	
**First Author:**	Pierre Lejeune
**First Author Secondary Information:**	
**Order of Authors:**	Pierre Lejeune

Powered by Editorial Manager® and ProduXion Manager® from Aries Systems Corporation

	Anthony Fratamico
	Frédéric Bouché
	Samuel Huerga-Fernández
	Pierre Tocquin
	Claire Périlleux, PhD
**Order of Authors Secondary Information:**	
**Additional Information:**	
**Question**	**Response**
Are you submitting this manuscript to a special series or article collection?	No
**Experimental design and statistics**<br><br>Full details of the experimental design and statistical methods used should be given in the Methods section, as detailed in our Minimum Standards Reporting Checklist. Information essential to interpreting the data presented should be made available in the figure legends.<br><br>Have you included all the information requested in your manuscript?	Yes
**Resources**<br><br>A description of all resources used, including antibodies, cell lines, animals and software tools, with enough information to allow them to be uniquely identified, should be included in the Methods section. Authors are strongly encouraged to cite Research Resource Identifiers (RRIDs) for antibodies, model organisms and tools, where possible.<br><br>Have you included the information requested as detailed in our Minimum Standards Reporting Checklist?	Yes
**Availability of data and materials**<br><br>All datasets and code on which the conclusions of the paper rely must be	Yes

either included in your submission or deposited in publicly available repositories (where available and ethically appropriate), referencing such data using a unique identifier in the references and in the "Availability of Data and Materials" section of your manuscript.<br><br>Have you have met the above requirement as detailed in our Minimum Standards Reporting Checklist?	

Powered by Editorial Manager® and ProduXion Manager® from Aries Systems Corporation

Manuscript Click here to access/download;Manuscript;Lejeune_et_al_GigaScience_v3.d

Led Color Gradient As A New Screening Tool For Rapid Phenotyping Of Plant Responses To Light Quality

Pierre Lejeune[1], Anthony Fratamico[2], Frédéric Bouché[1], Samuel Huerga-Fernández[1], Pierre Tocquin[1], Claire Périlleux[1]*

[1]InBioS - PhytoSYSTEMS, Laboratory of Plant Physiology, University of Liège, B22 Sart Tilman Campus, 4 Chemin de la Vallée, 4000 Liège, Belgium

[2]GDTech S.A. Avenue de l'Expansion, 7, B-4432 Alleur, Liège, Belgique

*To whom correspondence should be addressed. Tel: +32 4 3663833; Email: cperilleux@uliege.be

Abstract

Background

The increasing demand for local food production is fueling high interest in the development of controlled environment agriculture (CEA). In particular, LED technology brings energy-saving advantages together with the possibility to manipulate plant phenotypes through light quality control. However, optimizing light quality is required for each cultivated plant and specific purpose.

Findings

In this paper, we show that the combination of LED gradient setups with imaging-based non-destructive plant phenotyping constitutes an interesting new screening tool with the potential to improve speed, logistics, and information output. To validate this concept, an experiment was performed to evaluate the effects of a complete range of Red:Blue ratios on seven plant species: *Arabidopsis thaliana*, *Brachypodium distachyon*, *Euphorbia peplus*, *Ocimum basilicum*, *Oryza sativa*, *Solanum lycopersicum*, and *Setaria viridis*. Plants were exposed during four weeks to the light gradient and showed significant, but species-dependent, responses in terms of dimension, shape, and color. A time series analysis of phenotypic descriptors highlighted growth changes but also transient responses of plant shapes to the Red:Blue ratio.

Conclusion

This approach, which generated a large reusable dataset, can be adapted for addressing specific needs in crop production or fundamental questions in photobiology.

Keywords. LED, imaging, phenomics, light quality, light gradient, red blue ratio, controlled environment agriculture

Introduction

New urban agriculture business models are emerging as market demand for local production of high-quality fruits and vegetables is increasing [1]. This, in turn, is stimulating the development of techniques used in controlled environment agriculture, offering unique opportunities for year-round production independently of season, weather, soil conditions, or climate change, as well as reduced resource use and lower production costs [2,3].

The economic feasibility of CEA owes a lot to the development of the light-emitting diode (LED) technology, which progressively replaces traditional artificial lighting sources. Indeed, LED

lighting fixtures show a great potential for energy saving compared to former technologies (e.g. high-pressure sodium lamps) [4,5]. In addition, they provide control over spectral composition, flexible fixture format, durability, long operating lifetime, relatively cool emitting surfaces, and linear photon output with electrical input current [6]. These attributes can greatly facilitate the application of photobiology at all stages of crop production, from propagation to postharvest quality control. Besides providing energy for photosynthesis, light indeed plays a key role in many plant responses that depend on its duration, intensity, and spectrum, which are perceived by a battery of photoreceptors [7–9]. It can thus be expected that LED will revolutionize indoor crop production [5] as the technology is still improving in efficiency while capital costs keep decreasing [10].

Interestingly, CEA has its own breeding targets. Indeed, in addition to indoor-specific constraints (e.g. small size and short cycle), the desired plant's response to the environment resides in phenotypic plasticity rather than resilience to stress conditions [11]. For example, different light qualities could be used to grow the same lettuce genotype for different products such as green *versus* red salads [12], therefore genotypes that show such plasticity are desirable.

In the context of these fast technological developments, screening for CEA-specific breeding targets and optimizing environmental conditions for new business models are key steps. Meeting these needs efficiently requires high throughput approaches, such as those employed for plant phenomics [13]. Phenomics is a relatively recent research field, initially triggered by the huge demands for phenotyping capacity in functional genomics studies [14]. It has been focused primarily on model plants such as *Arabidopsis thaliana*, as well as major crops such as cereals and other large-scale productions. Phenomics rely heavily on imaging technologies that are non-destructive and allow the quantification of complex structures in a fast and highly repeatable way. Correlation between image-based descriptors and ground-truth data obtained by direct measurements has been demonstrated multiple times in different model systems. For example: i) projected leaf area or height have been shown to correlate well with direct

measurements of plant dimensions and biomass in wheat [15], *Arabidopsis* [16,17], or tomato [18], ii) geometric descriptors have been used to objectivize shape variations between *Arabidopsis* genotypes [19,20], iii) color indices based on simple RGB images have proven useful for discriminating differences in leaf chlorophyll content [21,22]. The requirement for high throughput phenotyping increases as the plant research community addresses the future challenges that agriculture will face with climate change [23]. Obviously, the same technological advances in sensors, imaging, automation, and data processing that benefit functional genomics can be used to evaluate plant phenotypes under indoor cultivation contexts as well as to identify either optimum conditions for available genotypes or fitter genotypes for indoor conditions.

A timely research investment for CEA development is thus to use plant phenomics to explore the many new avenues, constraints, and needs that currently emerge from the rapid worldwide adoption of LED technology. Previous studies aiming at evaluating the effects of light quality on plant production mostly compared limited numbers of discrete conditions (e.g. different ratios of Red:Blue, Red:Far-Red, %UV) within very specific combinations of target species/genotypes, environments, traits of interest, and phenotyping approaches [6]. As light sources and growing setups largely differ across laboratories, customizing the lighting conditions for each economically important plant remains complex, and knowledge gaps still limit the productivity of CEA [13]. Therefore, a more comprehensive method to characterize plant phenotypic responses to light quality is highly desirable and would also provide a boost to basic photobiology research in model systems.

In this paper, we examined the methodological advances provided by light quality gradients in terms of phenotyping speed, logistics, and information content, and whether this would facilitate studies of light quality responses. To our knowledge, light gradients have seldom been studied as such, except in agroecology contexts like forestry, where irradiance is the main variable factor [24–26]. Therefore, light quality gradients represent a new experimental

approach offering several potential advantages: i) a wide range of spectral ratios can be tested in one cycle, while all other parameters remain constant; ii) the continuous variation in light quality offers the possibility to detect thresholds, peaks, and downs in the plant response; iii) regressions can be used to estimate correlation, effect size, and significance in an easy and straightforward way; iv) when combined with non-destructive phenotyping methods such as time-series imaging, it provides detailed information on the plasticity of various target traits.

We designed a multi-species experiment to test a gradient of Red and Blue lights, since these colors have been the focus of many publications in the horticultural domain [6,27,28]. Smart LED luminaries were used to create a continuous range of Red:Blue ratios under otherwise controlled conditions, and an imaging platform was used to measure basic phenotypic traits related to growth, morphology and pigmentation of the plants (plant dimensions, shape factors, color indices). Among the numerous options for digital imaging setups that have been developed for a variety of applications and scientific questions [29], we opted for a simple low-cost design using off-the-shelf electromechanics, RGB cameras, and open-source image acquisition and analysis software. Depending on the purpose, such “maker-made” phenotyping stations can provide sufficient image quality and throughput as shown in a growing number of publications [30–32].

We chose to characterize seven plant species, based on their scientific and economical importance, as well as botanical and architectural diversity. We selected four dicot species: *Arabidopsis thaliana* (Brassicaceae), an obvious choice due to its importance in academic research and the wealth of genomic and phenomic knowledge, *Solanum lycopersicum* (Solanaceae) and *Ocimum basilicum* (Lamiaceae), two interesting models for horticultural applications, and *Euphorbia peplus* (Euphorbiaceae), a wild species studied for its medicinal properties. We also grew three monocot species (Poaceae): one temperate species, *Brachypodium distachyon*, one tropical crop, *Oryza sativa*, and one C4 wild species, *Setaria viridis*.

Results

Data description

Plants of seven different species were grown for 30 days under white light, then transferred to a gradient of Red to Blue LED lights for another 30 days, and finally returned to white light (Figure 1). Phenotypic data were collected twice a week from side- and top-view images. Image processing delivered three types of phenotypic descriptors: i) simple dimensions (e.g. height, width, projected area, fitted ellipse), ii) shape factors derived from dimensions (e.g. Roundness, Solidity, Circularity), iii) color density values (Red, Green, Blue, Hue, Saturation, Brightness), and their respective standard deviations. A detailed explanation of the phenotypic descriptors is provided in Table 1.

The effect of the gradient on each phenotypic descriptor was evaluated by calculating a linear regression against the log-transformed Red:Blue ratios measured at each plant location. Besides recording Pearson R and p-value, the slope and intercept of the regression were used to estimate descriptor values at both the minimal and the maximal Red:Blue ratios. The difference between these values was defined as the “effect size” of the gradient, which is expressed as the percentage difference across the Red:Blue gradient. The regression graphs can be generated for visualization using the R scripts provided in the “Code and data availability” section. These calculations were performed at each phenotyping time point in order to track the evolution of the Red:Blue ratio effects during and after the gradient treatment.

Species discrimination

The dataset described here allowed to evaluate the potential of the imaging platform to discriminate diverse plant species, from narrow-leaf monocots (*Brachypodium distachyon*, *Oryza sativa*, *Setaria viridis*) to large-leaf caulescent tomato (*Solanum lycopersicum*) or multi-plant bushes (*Euphorbia peplus, Ocimum basilicum*).

Figure 2 shows how species discrimination by principal component analysis performed, based on different combinations of the three types of phenotypic descriptors (dimensions, shape factors, color indices) and the two camera views (side- and top-view). As expected, the different species were best discriminated based on the full set of descriptors, all other combinations yielding only partial separations, especially for the three monocots. A main limitation was also found with *Arabidopsis thaliana*, whose basal rosette of flat leaves could only be characterized consistently from the top-view images.

Differential growth, shape, and color under variable Red:Blue ratio

In tomato (*S. lycopersicum*), which we take here as an example, plants were visually taller, wider, and bulkier as the Red:Blue ratio increased (Figure 3b,c). Capturing image-based phenotypic descriptors throughout the gradient treatment also revealed more subtle changes, such as a decrease in Circularity, a shape factor that quantifies area:perimeter variation (Figure 3d,e). This was likely due to the elongation of stems and petioles, which increased the convexities in the plant contours under high Red:Blue ratio. The Triangular Greenness Index (TGI) calculated from RGB density values also increased, indicating higher reflectance in the green broadband (Figure 3d,e). Since TGI was negatively correlated with chlorophyll content estimates (see supplemental Figure S1), this color change suggested a decrease in leaf chlorophyll content with higher Red:Blue ratios. This combination of phenotypes is consistent with previous studies showing that blue wavelengths reduce stem elongation and increase

chlorophyll concentration in *S. lycopersicum* [33,34]. Similar analyses were performed for the other six species. Figure 4 shows the calculated “effect sizes” of the Red:Blue gradient after 4 weeks of light treatment for 20 parameters that showed a highly significant correlation ($p<0.01$) with the Red:Blue ratio in at least one of the species. Although the effects on height and color described above for *S. lycopersicum* were mostly consistent across species, the pattern and amplitude of the effects on the full array of phenotypic descriptors appeared highly species-specific. For example, in *S. lycopersicum*, effects on dimension descriptors were observed in side-view images only, while in *E. peplus*, *B. distachyon*, and *O. sativa*, top-view dimensions were also affected, and in *A. thaliana*, *O. basilicum*, or *S. viridis,* no significant effects on any dimension descriptors were observed. In terms of shape and color descriptors, all species exhibited different patterns of responses to Red:Blue ratio, as sketched in Figure 5.

The increase in plant height with the Red:Blue ratio, observed in nearly all species studied here, is consistent with previous reports in horticultural literature involving phylogenetically distant eudicots such as cabbage [35], artichoke [36], cucumber [37], tomato [33,34], or lettuce [38]. Also, the greenness indices (GLI, TGI, or Chl-predicted) pointed towards a decrease in chlorophyll content with higher Red:Blue ratio, which is in line with reported effects of red and blue lights in lettuce [38], cabbage [35], tomato [34], cucumber, pepper, and radish [33]. There were two exceptions, however: i) the correlation between TGI and chlorophyll estimate was reversed in *O. sativa* (supplemental Figure S1), possibly as a consequence of leaf inclination and reflectance changes with light quality, and ii) in *S. viridis,* the only representative of C4 plants in this study, there was no correlation between TGI and chlorophyll estimates and the effect of the Red:Blue ratio on chlorophyll was opposite to the other species. These cases are good reminders that chlorophyll content is not always the main explanatory variable in a color index. Indeed, although RGB reflectance was shown to provide estimation of chlorophyll contents in different species [21,39,40], it lacks specificity and is sensitive to other pigments as well as to leaf texture and/or inclination.

Patterns of change over time

The effect size of the Red:Blue gradient was found to change over time. In tomato, it was strongest 2 weeks after the start of the treatment for a number of descriptors (Figure 3d) but diminished markedly afterwards, suggesting a possible acclimation process. The same analysis performed in the other species showed both similarities and species-specific trends (Figure 6). For example, in *E. peplus, B. distachyon,* and *O. sativa,* the effect size increased with time and then diminished slowly after return to white light. In *O. basilicum,* we did not observe any significant effect during exposure to the gradient, but the transfer back to white light caused sudden and transient changes in parameters such as Height, Circularity and TGI.

Conclusion

It is clear from the preceding section that the effects of light quality on plant phenotypes are strongly species-dependent, so that no predictive clues can be generalized. Experimentation is thus absolutely required before any application of LED technology in CEA or other research contexts. Here, we demonstrate that, compared to discrete conditions, the use of a light gradient allows capturing both obvious and inconspicuous phenotypic effects while avoiding the interference of other environmental variations, which still hampers comparisons of data acquired in different plant growth facilities. The analysis of our dataset also shows that high throughput phenotyping is required to capture the complexity of plant plasticity and that a time course is needed to measure possible transient effects.

Additional features could improve the platform described here. For instance, throughput could be increased by adding automated steps (plant conveyors or moving top-view cameras on gantry) that were not included in our maker-made platform but are quite common for higher capacity facilities [41–43]. The accuracy, relevance, and depth of imaging could be improved by using new technologies such as spectral, tridimensional, thermal, or fluorescence cameras, depending on the desired application and/or traits of interest. In particular, this would address

the pertinence of the RGB color indices and the biases caused by plant shapes and leaf inclination, which were also reported in studies on spectral imaging [44]. Image analysis could be accomplished with other available softwares, some of which offer more specialized functionalities than the free and popular generalist package, ImageJ, which was used here. The online resource http://www.plant-image-analysis.org curates currently available tools for morphological plant image analysis [45].

More elaborate data processing could also be explored beyond linear regression, while machine learning approaches could facilitate the interpretation of the complex set of parameters generated by imaging, especially when it comes to phenotypic descriptors such as color indices and shape factors. For example, classification techniques would allow categorizing plants according to predefined criteria and provide the user of the dataset with a more holistic understanding of the plant phenotype.

All these perspectives further broaden the potential advantages of combining LED gradients with imaging-based plant phenotyping for both environmental optimization and genotypic selection of CEA targets. The methodology can be adapted to multiple use-cases by changing the LED wavelengths, the gradient configurations, and the timing of the light treatments.

Methods

Plant materials

Arabidopsis thaliana Col-0 seeds were obtained from a public seedbank (NASC, Nottingham, UK) and *Brachypodium distachyon* Bd21-3 seeds from Prof. R. Amasino (University of Wisconsin, USA). Seeds of *Euphorbia peplus* were obtained from fairdinkumseeds.com (Queensland, Australia). Seeds of *Setaria viridis* A10.1 were obtained from USDA Iowa State University Agricultural Research Service (Ames, IO, USA). Seeds of *Solanum lycopersicum* cv Ailsa Craig were obtained from TGRC (Davis, CA, USA). *Ocimum basilicum* cv Genovese

seeds were obtained from Le Jardin de Bellecourt (Bellecourt, Belgium). Seeds of *Oryza sativa cv.* Nipponbare were obtained from IRRI (Los Baños, Laguna, The Philippines). All materials were obtained and used within the Rights and Obligations of the Recipient as specified by the International Treaty on Plant Genetic Resources for Food and Agriculture adopted by the FAO Conference on 3 November 2001 and entered into force on 29 june 2004.

Growth conditions

Seeds were sown in 4.5 cm fiber pots (Jiffypots®, Jiffy, Zwijndrecht, The Netherlands) filled with a 4:1 mix of leaf mould and baked clay granules. The fiber pots were placed on 120 x 18 x 14 cm cultivation gutters (Goponic, Nouméa, France) and irrigated by capillarity through a wet cultivation felt mat (Feutriplanta®, Jardirama, Warsage, Belgium) and wicks dipping in the water (Figure 1a and 1b). The gutters were placed for 30 days in a Conviron PGV36 growth room (Conviron, Winnipeg, Canada) at 21°C day/night, 70% relative humidity, 12-h photoperiod, at an irradiance of ± 130-150 $\mu E.m^{-2}.s^{-1}$ provided by Sylvania Luxline Plus T5 FHO 54W tubes (Osram-Sylvania, Wilmington, MA, USA) delivering 4000K white light. After 30 days, Jiffypots were transplanted into 12-cm square plastic pots filled with the same substrate, supplemented with 6 $gr.L^{-1}$ of slow release fertilizer (Osmocote Exact Standard 5-6 M, ICL Specialty Fertilizers). Only one plant per pot was kept, except for *E. peplus* (6 plants/pot) and *O. basilicum* (up to 9 plants/pot) to account for their usual mode of cultivation in bushes. The pots were fitted at the bottom with a 2 x 10 cm felt wick for capillarity irrigation and randomly placed on the deck of the cultivation gutters. The gutters were then placed in the same environmental conditions than previously, except for the lighting which was provided either by white fluorescent tubes as before or by adjustable Lumiatec LED luminaries providing a range of Red:Blue light ratios (described below and Figure 1c). CO_2 concentration was ambient and remained within 390-410 ppm throughout the experiment (measurements performed with IRGA analyzer WMA-5 PP Systems, Amesbury, MA, USA).

Layout under Red:Blue gradient: Each room (3 m²) allowed 12 gutters of 10 pots (Figure 1c). The placement of the plants was organized in rows and columns so that each pot could be registered by Room:Row:Column coordinates and labelled with a unique QR-code. The gutters corresponded to the columns, and there were 36 pots per species in 3 contiguous rows of 12 pots, except for *Arabidopsis* that had 4 rows, so 48 pots. No guard rows were used. At the beginning of the LED gradient treatment, a randomization step was performed for each species within their block, after which the pots were kept at the same position until the end of the treatment. On day 60, the plants were all transferred back to white light conditions, and the experiment was stopped after 4 weeks.

Red-Blue light gradient

The phytotronic cabinets were equipped with 15 Lumiatec PHS :: 16 (300W) luminaries (GDTech, Alleur, Belgium) each. These luminaries are controllable over 16 channels and fitted with PCB-LEDs offering 2 x 6 Blue LED 455 nm, 6 x 6 White LED 4000K, 1 x 6 Green LED 520 nm, 1 x 6 Yellow LED 593 nm, 2 x 6 Red LED 635 nm, 2 x 6 Hi-Red LED 660 nm, 1 x 6 far-red LED 730 nm, and 1 x 6 UV LED 280 nm. The 15 luminaries were regularly distributed at a distance of 0.5 m (Figure 1c) and were controlled per cluster of 3 using the Lumiatec control interface. The Blue and Hi-Red channels were adjusted as shown in Figure 1c in order to create a gradient of Red:Blue ratio (Figure 1d). The light spectrum and intensity across the growth chambers were monitored using a HiPoint HR-550 spectrophotometer (TAIWAN HIPOINT CORP., Kaohsiung, Taiwan). PPFD under the gradient conditions was 100-150 $\mu E.m^{-2}.s^{-1}$ (Figure 1e).

Imaging hardware

Plants grown in individual pots were placed on a rotating platform and photographed laterally (6 side-view images during a 180° rotation) or from the ceiling (1 top-view image). The imaging setup was built with aluminium profiles supporting white diffusive PVC walls. The cabinet was

illuminated by 25 x 25 cm white light LED panels (Araponics, Liège, Belgium). Lighting was optimised for taking pictures with a diffusive back-lit white background for side-view images and a black cloth background for top-view images. A step-motor platform was used to rotate the pot while two CMOS RGB 12 Mpx cameras (Dalsa Genie-nano 4040, Dalsa, Waterloo, Canada) acquired plant images from side and top view and one color HD webcam (Logitech, Lausanne, Switzerland) read QR-coded labels on the pot. Spatial specifications allowing to reproduce this setup are provided in supplemental Figure S2. The Genie-Nano cameras were fitted with high resolution 25-mm focal length Tamron M111FM25 lenses, which allowed to image plants up to 150-cm high and 100-cm wide with an estimated smallest detail size of +/- 0.5 mm at a working distance of 200 cm, based on sensor dimensions (14.2 x 10.4 mm, 4112 x 3008 pixels) and lens optical resolution (3.1 µm "pixel pitch"). Diaphragm closure of the lenses was set to F8.0, exposure time to 0.2 msec and gain to 6. The cameras and the stepper-motor were controlled through a dedicated software written in Python and running on a Linux computer in order to synchronize plant identification, rotation, and image acquisition. The adjustment of basic camera settings (e.g. shutter speed, gain, output format, …) used libraries from OpenCV (https://pypi.org/project/opencv-python/) and Aravis (https://github.com/AravisProject/aravis), while rotation functionalities (i.e. speed, number and time of acquisitions after QR-code detection) were programmed by us. Typically, six side-view images and one top-view image were acquired during a 180° rotation in 4 seconds (45° per sec.). The pots were manually loaded on the rotating platform through a sliding door. After rotation was initiated, the imaging cycle started when the QR-code identifier was read by the webcam, and each image acquired by the Genie-Nano cameras was saved under a unique ID. The complete imaging cycle was about 10 seconds per pot.

Image processing and generation of phenotypic descriptors

An automated script was developed using the macro language of the ImageJ open source package (Fiji distribution) [46] in order to extract plant phenotypic descriptors from each image.

The successive steps were: i) reading the raw image in bayer format, ii) getting metadata, e.g. date, pot ID, camera view, frame nr, iii) white balance and spatial calibration based on a reference color chart, iv) segmentation of the plant from background using grey-scale or color thresholding, v) measurement of plant dimensions and shape factors, vi) extraction of color components in either RGB or HSB color space, vii) exporting raw data in text format (.csv). R version 3.6.1 for macOSX (available at https://cran.r-project.org/bin/macosx/) running under Rstudio version 1.3.1093 (Rstudio, Boston, MA, USA) was used to : i) compute additional shape factors as ratios from existing measurements, such as Voxel, Compactness, Anisotropy; ii) compute color indices such as Green Leaf Index (GLI), and Triangular Greenness Index (TGI); iii) generate a chlorophyll content prediction based on RGB values; iv) generate scatter plots to visually check for abnormal measurements due to e.g. corrupted images, before further statistical use; v) aggregate the multiple camera measurements per pot (e.g. the side view camera generated 6 images from which the mean, max, min and median values were computed); vi) merge imaging data with plant metadata (species, spatial location, intensity and quality of light at plant location, estimated chlorophyll content). A more detailed description of the image processing is found in supplemental Table S1.

Chlorophyll content estimate

The leaf chlorophyll content was estimated with a hand-held probe measuring the transmittance ratio of cell walls at 931nm versus chlorophyll at 653 nm (Apogee MC-100, Apogee Instruments, Logan, UT, USA). These measurements were performed once, at the end of the Red:Blue gradient treatment. Species-specific calibration models provided with the instrument were used for tomato and rice, whereas a generic model, averaged from multiple species (described in https://www.apogeeinstruments.com/content/MC-100-manual.pdf) was used for the other species. At least 6 measurements were made on minimum 3 different mature leaves per pot. The measurements were averaged per pot.

Source Code and Data Availability

Raw images, image analysis script, and raw data file are available at zenodo.org [47].

R script for statistical analysis, subsequent processed data files and plot files are available at codeocean.com [48].

Abbreviations.

CEA: Controlled Environment Agriculture; GLI: Green Leaf Index; LED: Light Emitting Diode; PCA: Principal Component Analysis; PCB: Printed Circuit Board; PFD: photon flux density; PPFD: photosynthetic photon flux density; QR-code: Quick Response code; RGB: Red Green Blue; TGI: Triangular Greenness Index

Acknowledgements

General:

Early access to the Lumiatec LED lighting systems was possible with the collaboration of GDTech and Araponics R&D teams, especially George Ferdinand, Michaël Menu, Julien Reuland and Dylan Dohogne. The authors are also grateful to Sébastien Steyaert and Gabriel Berger, for their technical assistance for plant cultivation and imaging, and to Profs Frédéric Lebeau and Guillaume Lobet for fruitful discussions and comments on the manuscript.

Author contributions:

PL, AF, FB, SHF, PT and CP designed the experiment and wrote the paper. AF and PT set-up the Lumiatec luminaries. PL created the imaging cabinet; AF and PT developed the image

acquisition software and interface. PL designed the image analysis script and processed the raw data.

Funding:

This research was supported by the European Union and the Walloon Region of Belgium, via the European Funds for Regional Development 2014-2020 / En Mieux (Tropical Plant Factory portfolio, Project C Plant'HP) and the Competitiveness cluster Wagralim (Project VeLiRe). Frédéric Bouché is an FNRS post-doc fellow (FC87200) and Samuel Huerga Fernández has an FNRS-FRIA grant (FC21283).

Competing interests:

The authors declare that there is no conflict of interest regarding the publication of this article.

References

1. Gómez C, Currey CJ, Dickson RW, Kim H-J, Hernández R, Sabeh NC, et al.. Controlled Environment Food Production for Urban Agriculture. *HortScience*. 2019; doi: 10.21273/HORTSCI14073-19.
2. Kozai T. Plant Factories with Artificial Lighting (PFALs): Benefits, Problems, and Challenges. In: Kozai T, editor. *Smart Plant Fact*. Singapore: Springer Singapore; 2018. p. 15-29.
3. SharathKumar M, Heuvelink E, Marcelis LFM. Vertical Farming: Moving from Genetic to Environmental Modification. *Trends Plant Sci*. 2020; doi: 10.1016/j.tplants.2020.05.012.
4. Cocetta G, Casciani D, Bulgari R, Musante F, Kołton A, Rossi M, et al.. Light use efficiency for vegetables production in protected and indoor environments. *Eur Phys J Plus*. 2017; doi: 10.1140/epjp/i2017-11298-x.
5. Mitchell CA, Sheibani F. Chapter 10 - LED advancements for plant-factory artificial

lighting. In: Kozai T, Niu G, Takagaki M, editors. Plant Factory (Second Edition). Academic Press; 2020. p. 167-184.

6. Bantis F, Smirnakou S, Ouzounis T, Koukounaras A, Ntagkas N, Radoglou K. Current status and recent achievements in the field of horticulture with the use of light-emitting diodes (LEDs). *Sci Hortic*. 2018; doi: 10.1016/j.scienta.2018.02.058.

7. Paik I, Huq E. Plant photoreceptors: Multi-functional sensory proteins and their signaling networks. *Semin Cell Dev Biol*. 2019; doi: 10.1016/j.semcdb.2019.03.007.

8. Chory J. Light signal transduction: an infinite spectrum of possibilities. *Plant J*. 2010; doi: 10.1111/j.1365-313X.2009.04105.x.

9. Davis PA, Burns C. Photobiology in protected horticulture. *Food Energy Secur*. 2016; doi: 10.1002/fes3.97.

10. Kusuma P, Pattison PM, Bugbee B. From physics to fixtures to food: current and potential LED efficacy. *Hortic Res*. 2020; doi: 10.1038/s41438-020-0283-7.

11. Folta KM. Breeding new varieties for controlled environments. *Plant Biol*. 2019; doi: 10.1111/plb.12914.

12. Owen WG, Lopez RG. End-of-production Supplemental Lighting with Red and Blue Light-emitting Diodes (LEDs) Influences Red Pigmentation of Four Lettuce Varieties. *HortScience*. 2015; doi: 10.21273/HORTSCI.50.5.676.

13. Marondedze C, Liu X, Huang S, Wong C, Zhou X, Pan X, et al.. Towards a tailored indoor horticulture: a functional genomics guided phenotypic approach. *Hortic Res*. 2018; doi: 10.1038/s41438-018-0065-7.

14. Ninomiya S, Baret F, Cheng Z-M (Max). Plant Phenomics: Emerging Transdisciplinary Science. *Plant Phenomics*. 2019; doi: 10.34133/2019/2765120.

15. Golzarian MR, Frick RA, Rajendran K, Berger B, Roy S, Tester M, et al.. Accurate inference of shoot biomass from high-throughput images of cereal plants. *Plant Methods*. 2011; doi: 10.1186/1746-4811-7-2.

16. Vasseur F, Bresson J, Wang G, Schwab R, Weigel D. Image-based methods for phenotyping growth dynamics and fitness components in Arabidopsis thaliana. *Plant*

Methods. 2018; doi: 10.1186/s13007-018-0331-6.

17. Arend D, Lange M, Pape J-M, Weigelt-Fischer K, Arana-Ceballos F, Mücke I, et al.. Quantitative monitoring of Arabidopsis thaliana growth and development using high-throughput plant phenotyping. *Sci Data*. 2016; doi: 10.1038/sdata.2016.55.
18. Laxman RH, Hemamalini P, Bhatt RM, Sadashiva AT. Non-invasive quantification of tomato (Solanum lycopersicum L.) plant biomass through digital imaging using phenomics platform. *Indian J Plant Physiol*. Springer Verlag; 2018; doi: 10.1007/s40502-018-0374-8.
19. Camargo A, Papadopoulou D, Spyropoulou Z, Vlachonasios K, Doonan JH, Gay AP. Objective Definition of Rosette Shape Variation Using a Combined Computer Vision and Data Mining Approach. Candela H, editor. *PLoS ONE*. 2014; doi: 10.1371/journal.pone.0096889.
20. De Vylder J, Vandenbussche F, Hu Y, Philips W, Van Der Straeten D. Rosette Tracker: An Open Source Image Analysis Tool for Automatic Quantification of Genotype Effects. *Plant Physiol*. 2012; doi: 10.1104/pp.112.202762.
21. Vollmann J, Walter H, Sato T, Schweiger P. Digital image analysis and chlorophyll metering for phenotyping the effects of nodulation in soybean. *Comput Electron Agric*. 2011; doi: 10.1016/j.compag.2010.11.003.
22. Hunt ER, Doraiswamy PC, McMurtrey JE, Daughtry CST, Perry EM, Akhmedov B. A visible band index for remote sensing leaf chlorophyll content at the canopy scale. *Int J Appl Earth Obs Geoinformation*. 2013; doi: 10.1016/j.jag.2012.07.020.
23. Pieruschka R, Schurr U. Plant Phenotyping: Past, Present, and Future. *Plant Phenomics*. 2019; doi: 10.34133/2019/7507131.
24. Poorter L. Growth Responses of 15 Rain-Forest Tree Species to a Light Gradient: The Relative Importance of Morphological and Physiological Traits. *Funct Ecol. 1999*; 13:396-410.
25. Kelly J, Jose S, Nichols JD, Bristow M. Growth and physiological response of six Australian rainforest tree species to a light gradient. *For Ecol Manag*. 2009; doi:

10.1016/j.foreco.2008.09.008.

26. Cheng X, Yu M, Wang G, Wu T, Zhang C. Growth, Morphology And Biomass Allocation In Response To Light Gradient In Five Subtropical Evergreen Broadleaved Tree Seedlings. *J Trop For Sci*. Forest Research Institute Malaysia; 25:537–462013;

27. Ouzounis T, Rosenqvist E, Ottosen C-O. Spectral Effects of Artificial Light on Plant Physiology and Secondary Metabolism: A Review. *HortScience*. 2015; doi: 10.21273/HORTSCI.50.8.1128.

28. Jishi T. LED Lighting Technique to Control Plant Growth and Morphology. In: Kozai T, editor. *Smart Plant Fact*. Singapore: Springer Singapore; 2018. p. 211-222.

29. Zhang Y, Zhang N. Imaging technologies for plant high-throughput phenotyping: a review. *Front Agric Sci Eng*. 2018; doi: 10.15302/J-FASE-2018242.

30. Tsaftaris SA, Noutsos C. Plant Phenotyping with Low Cost Digital Cameras and Image Analytics. In: Athanasiadis IN, Rizzoli AE, Mitkas PA, Gómez JM, editors. *Inf Technol Environ Eng*. Berlin, Heidelberg: Springer Berlin Heidelberg; 2009. p. 238-251.

31. Fahlgren N, Gehan MA, Baxter I. Lights, camera, action: high-throughput plant phenotyping is ready for a close-up. *Curr Opin Plant Biol*. 2015; doi: 10.1016/j.pbi.2015.02.006.

32. Lien MR, Barker RJ, Ye Z, Westphall MH, Gao R, Singh A, et al.. A low-cost and open-source platform for automated imaging. *Plant Methods*. 2019; doi: 10.1186/s13007-019-0392-1.

33. Snowden MC, Cope KR, Bugbee B. Sensitivity of Seven Diverse Species to Blue and Green Light: Interactions with Photon Flux. Ezura H, editor. *PLOS ONE*. 2016; doi: 10.1371/journal.pone.0163121.

34. Dieleman JA, De Visser PHB, Meinen E, Grit JG, Dueck TA. Integrating Morphological and Physiological Responses of Tomato Plants to Light Quality to the Crop Level by 3D Modeling. *Front Plant Sci*. 2019; doi: 10.3389/fpls.2019.00839.

35. Fan X, Zang J, Xu Z, Guo S, Jiao X, Liu X, et al.. Effects of different light quality on growth, chlorophyll concentration and chlorophyll biosynthesis precursors of non-heading

Chinese cabbage (Brassica campestris L.). *Acta Physiol Plant*. 2013; doi: 10.1007/s11738-013-1304-z.

36. Rabara RC, Behrman G, Timbol T, Rushton PJ. Effect of Spectral Quality of Monochromatic LED Lights on the Growth of Artichoke Seedlings. *Front Plant Sci*. 2017; doi: 10.3389/fpls.2017.00190.
37. Hernández R, Kubota C. Physiological responses of cucumber seedlings under different blue and red photon flux ratios using LEDs. *Environ Exp Bot*. 2016; doi: 10.1016/j.envexpbot.2015.04.001.
38. Dougher TAO, Bugbee B. Differences in the Response of Wheat, Soybean and Lettuce to Reduced Blue Radiation¶. *Photochem Photobiol*. 2007; doi: 10.1562/0031-8655(2001)0730199DITROW2.0.CO2.
39. Hunt ER, Daughtry CST, Eitel JUH, Long DS. Remote Sensing Leaf Chlorophyll Content Using a Visible Band Index. *Agron J*. 2011; doi: 10.2134/agronj2010.0395.
40. Gracia-Romero A, Kefauver SC, Vergara-Díaz O, Zaman-Allah MA, Prasanna BM, Cairns JE, et al.. Comparative Performance of Ground vs. Aerially Assessed RGB and Multispectral Indices for Early-Growth Evaluation of Maize Performance under Phosphorus Fertilization. *Front Plant Sci*. 2017; doi: 10.3389/fpls.2017.02004.
41. Reuzeau C, Frankard V, Hatzfeld Y, Sanz A, Van Camp W, Lejeune P, et al.. Traitmill™: a functional genomics platform for the phenotypic analysis of cereals. *Plant Genet Resour*. 2006; doi: 10.1079/PGR2005104.
42. Fiorani F, Schurr U. Future Scenarios for Plant Phenotyping. *Annu Rev Plant Biol*. Annual Reviews; 2013; doi: 10.1146/annurev-arplant-050312-120137.
43. Li Z, Guo R, Li M, Chen Y, Li G. A review of computer vision technologies for plant phenotyping. *Comput Electron Agric*. 2020; doi: 10.1016/j.compag.2020.105672.
44. Paulus S, Mahlein A-K. Technical workflows for hyperspectral plant image assessment and processing on the greenhouse and laboratory scale. *GigaScience*. 2020; doi: 10.1093/gigascience/giaa090.
45. Lobet G, Draye X, Périlleux C. An online database for plant image analysis software

tools. *Plant Methods*. 2013; doi: 10.1186/1746-4811-9-38.

46. Schneider CA, Rasband WS, Eliceiri KW. NIH Image to ImageJ: 25 years of image analysis. *Nat Methods*. 2012; doi: 10.1038/nmeth.2089.

47. Lejeune P, Fratamico A, Bouché F, Huerga-Fernández S, Tocquin P, Périlleux C. Data and scripts used in the paper entitled “Led Color Gradient As A New Screening Tool For Rapid Phenotyping Of Plant Responses To Light Quality” by Pierre LEJEUNE et al. *Zenodo*. 2021; doi: 10.5281/zenodo.4071810.

48. Lejeune P, Fratamico A. Data and R script used in the paper entitled “Led Color Gradient As A New Screening Tool For Rapid Phenotyping Of Plant Responses To Light Quality” by Pierre LEJEUNE et al. *Code Ocean*. 2021; doi: 10.24433/CO.6400538.v1.

Figures and Tables

Figure 1.

Cultivation setup under Red:Blue light gradient. (a) 30-day-old plantlets at the end of pre-cultivation period. (b) Cultivation system before (small pots) and after (large pots) transfer under the Lumiatec LED luminaries. (c) Red:Blue gradient setup. Arrangement and setting of the 5 clusters of LED luminaries in the phytotronic cabinet. (d) Red:Blue ratio measured at each plant position, PFD = photon flux density. (e) Total light irradiance measured across the gradient, PPFD = Photosynthetic PFD.

Figure 2.

Principal components analysis discrimination of seven species based on various selections of measured parameters. Species color codes in panel (a). Imaging data collected over 3 timepoints between 21 and 29 days after transfer under Red:Blue gradient were used.

Figure 3.

Example of plant phenotypes in *S. lycopersicum* under the Red:Blue gradient. (a) light gradient. (b) side-view and (c) top-view images of a row of tomato plants 21 days after transfer to the gradient conditions. (d) Use of linear regression to estimate correlation (R^2), significance (p-value), and "effect size" (% difference across the gradient) for some descriptors. (e) Evolution of the gradient “effect size” as a function of time for the same descriptors as in (d).

Figure 4.

Effect size of the Red:Blue gradient (% difference between 100% Red and 100% Blue), estimated 29 days after the start of the gradient. The significance categories are based on the p-value of the computed R^2 . Side-view data for *A. thaliana* are not shown (NA).

Figure 5.

Schematic representation of the phenotypic variations caused by a Red:Blue light gradient in seven plant species. Effects observed 29 days after the start of the light gradient.

Figure 6.

Time-course evolution of the"effect size"of the Red:Blue gradient for 3 phenotypic descriptors in 6 species. Vertical dotted line: end of the Red:Blue gradient treatment and return to white light. The significance categories are based on the p-value of the computed R^2. Side-view data for *A. thaliana* are not shown (NA).

Table 1.

Plant dimension, shape, and color parameters measured by imaging: definition, calculation, and units.

**Label**	**Definition**	**Formula**	**Unit or scale**
		**Dimensions**	
Side-view HeightMax	Maximum height out of 6 side-view images during 180° rotation		mm
Side-view WidthMax	Maximum width out of 6 side-view images during 180° rotation		mm
Side-view AreaMean	Mean Projected Area out of 6 side-view images during 180° rotation		mm^2
Top-view Area	Projected Area out of 1 top view image		mm^2
Top-view MeanFeret	Average of maximum and minimum distances between 2 points along the selection boundary.		mm
Voxel	Plant volume estimate combining side- and top-view area of the plant	sqroot (max(side-view area) * min(side-view area) * top-view area)	mm^3
		**Shape factors**	
Side- and Top-view Roundness	Degree of similarity to a circle derived from the fitted ellipse axes	minor axis / major axis (of the fitted ellipse)	Scale 0 to 1
Side- and Top-view Solidity	Overall concavity derived from area and convex-hull measurements	area / convex-hull area	Scale 0 to 1
Side- and Top-view Convexity	Edge "roughness" derived from convex hull and perimeter measurements	convex-hull perimeter / perimeter	Scale 0 to 1
Side- and Top-view Circularity	Ratio of the area of the shape to the area of a circle having the same perimeter (a.k.a "isoperimetric quotient")	4π * area / perimeter^2	Scale 0 to 1

Side- and Top-view Compactness	Degree of compacity derived from the ratio of the diameter a circle with the same area to the major axis of the fitted ellipse	sqroot((4/π) * area) / major ellipse axis	Scale 0 to 1
**Color indices**			
Side- and Top-view HueMean	Average hue component of the plant's color after transformation of the RGB image into HSB model (Hue Saturation Brightness)		Scale 0 to 255
Side- and Top-view HueCv	Coefficient of variation (CV) of the plant's pixels hue	stdev(hue) / avg(hue)*100	%
Side- and Top-view SaturationMean	Average saturation component of the plant's color after transformation of the RGB image into HSB model		Scale 0 to 255
Side- and Top-view BrightnessMean	Average brightness component of the plant's color after transformation of the RGB image into HSB model		Scale 0 to 255
Side- and Top-view RedMean	Average red component of the plant's color in the RGB model		Scale 0 to 255
Side- and Top-view GreenMean	Average green component of the plant's color in the RGB model		Scale 0 to 255
Side- and Top-view BlueMean	Average blue component of the plant's color in the RGB model		Scale 0 to 255
Side- and Top-view Density	Integrated density: The sum of the grey values of the pixels in the image or selection	area * mean grey value	
Top-view GLI	Green Leaf Index : vegetation index for use with a digital RGB camera	(2 * green - red - blue) / (2 * green + red + blue)	
Top-view TGI	Triangular Greenness Index : approximate area of a triangle bounding a leaf reflectance spectrum, where the vertices are in the red, green, and blue wavelengths.	((670 - 480) * (red - green) - (670 - 550)*(red - blue)) / -200	
Top-view Chl_predicted	Predicted leaf chlorophyll content derived from multiple linear regression using Red Green and Blue components of the plant color in the RGB model	440 + blue * 7.266 + red * 10.873 + green * -15.545	µmoles/m²

Supplementary materials

Figure S1.

Correlation between leaf chlorophyll content, as estimated manually with an Apogee MC-100 chlorophyll meter, and the Triangular Greenness Index (TGI) computed from RGB images.

Figure S2.

Blueprint of the imaging cabinet.

Table S1.

Steps in the image processing to generate plant shape and color proxies.

Nr	Description	Comment
**Steps in ImageJ**		
1	Read image file, get plant name, camera view, and frame number	"_0_" = side view "_1_" = top view
2	Find blue square in the color target and extract x,y coordinates	Convert to HSB color model, threshold light blue objects and record x,y coordinates
3	White balance using grey values on the reference target card	Described by V. Bindokas, modified by P. Mascalchi, and adapted by us. https://github.com/pmascalchi/ImageJ_Auto-white-balance-correction
4	Set the ROI (region of interest)	To remove borders and reference card
5	Create a HSB (hue, saturation, intensity) image	The HSB image is used later for color measurements
6	Separate RGB channels into 3 grey-level images	For both thresholding and computing greenness indices
7	Side-view images only:<br>Threshold on the B (blue) channel	To segment the plant from the background before measurements
8	Top-view images only:<br>Color threshold in HSB (hue, saturation, brightness) color space	Color thresholding is much slower than single channel thresholding, but is necessary when the background is not uniform as is the case with top view images
9	Eliminate small noise blobs based on size threshold	To eliminate any small background artifacts
10	Erode irregularities around the segmented object shape	To increase precision of contour measurements
11	Create a selection for morphological measurements	This is a binary “mask” of the plant
12	Save cropped color image for visual check	For rapid post-processing visual checks the smallest

		region enclosing the plant is saved as a separate color image
13	Measure plant dimensions on the segmented shape	"Basic" morphology parameters including : area, perimeter, height, width, major and minor axis lengths and angles, bounding box, centroid, solidity, circularity, aspect ratio, roundness
14	Compute convex hull area and perimeter	Useful for computing convexity indices
15	Save hull image	For rapid post-processing visual checks if needed
16	Redo a more stringent threshold to remove mixed background/plant pixels	The 2-3 pixels in the perimeter of the shape are a mix of background and plant color, and therefore need to be removed before measuring plant color parameters
17	Erode the borders of the plant to eliminate the edge pixels	To further remove mixed color pixels
18	Create a reduced mask based on the stringent threshold	To be applied on the RGB and HSB separated channels
19	Measure Red, Green, and Blue, densities on the reduced mask	The reduced mask is applied on each of the previously splitted R, G, and B channels. Measurements include mean density, stdev, mode, min, and max values
20	Measure Hue, Saturation, and Brightness densities on the reduced mask	The reduced mask is applied on each of the hue, saturation, and brightness channels. Measurements include mean density, stdev, mode, min, max, skewness, and kurtosis values
21	Export data to text file	All morphology and color measurements are saved in a csv file for further statistical analysis
	**Steps in Rstudio**	
22	Extract metadata from the filenames	Get Plant id, camera id, date:time in separate fields
23	Merge image and plant data	Get Species, Room, and Location of each plant from a separate plant file

24	Compute days after sowing (DAS) and days under gradient conditions for each imaging time point	
25	Perform visual quality check by plotting dimensions and color indices	For each species and time point, plotting Height *vs* Width indicates if there are clear abnormal measurements due e.g. to objects in the background.
26	Flag clear outliers	Outliers are flagged based on step 25 and on color measurements of the background reference card
27	Aggregate image data per plant	The measurements from the 6 side-view images are aggregated into one value per plant. For ex. Side area is the average of 6 images, side height and width are the maximum values.<br>Top- and side-view measurements are aggregated per plant and timepoint
28	Compute additional derived measurements	Voxel, Verticality, Green Leaf Index, Triangular Greenness Index, Chlorophyll content prediction are calculated
29	Merge imaging data and light mapping data	The local light data (intensity, spectra, computed Red:Blue ratio and phytochrome photostationary state (PSS)) is merged with plant imaging data
30	Merge imaging data and manual measurement data	E.g. leaf chlorophyll content recorded with Apogee probe

Code Ocean description:

The script “Data Analysis.R” performs linear regressions in order to describe the effect of local light composition (Red:Blue ratio) on a selection of phenotypic descriptors recorded by image analysis from plants of seven species growing under a gradient of light color (from Blue to Red). The script generates individual regression plots per each species / phenotypic descriptor / timepoint, as well as the time course plots of the gradient effect (calculated as described below) per each species / phenotypic descriptor.

The seven plant species were grown for 30 days under the Red:Blue gradient then returned

to standard white light. Phenotypic data were collected twice a week from side- and top-view images. Image processing delivered three types of phenotypic descriptors: i) simple dimensions (e.g. height, width, projected area, fitted ellipse), ii) shape factors derived from dimensions (e.g. Roundness, Solidity, Circularity), iii) color density values (Red, Green, Blue, Hue, Saturation, Brightness), and their respective standard deviations. The effect of the gradient on each phenotypic descriptor was evaluated by calculating a linear regression against the log-transformed Red:Blue ratios measured at each plant location. Besides recording Pearson R and p-value, the slope and intercept of the regression were used to estimate descriptor values at both the minimal and the maximal Red:Blue ratios. The difference between these values was defined as the "effect size" of the gradient, which is expressed as the percentage difference across the Red:Blue gradient. These calculations were performed at each phenotyping time point in order to track the evolution of "effect size" during and after the gradient treatment.

Figure1_Cultivation_setup

Click here to access/download;Figure;Figure1_Cultivation_setup.pdf.pdf

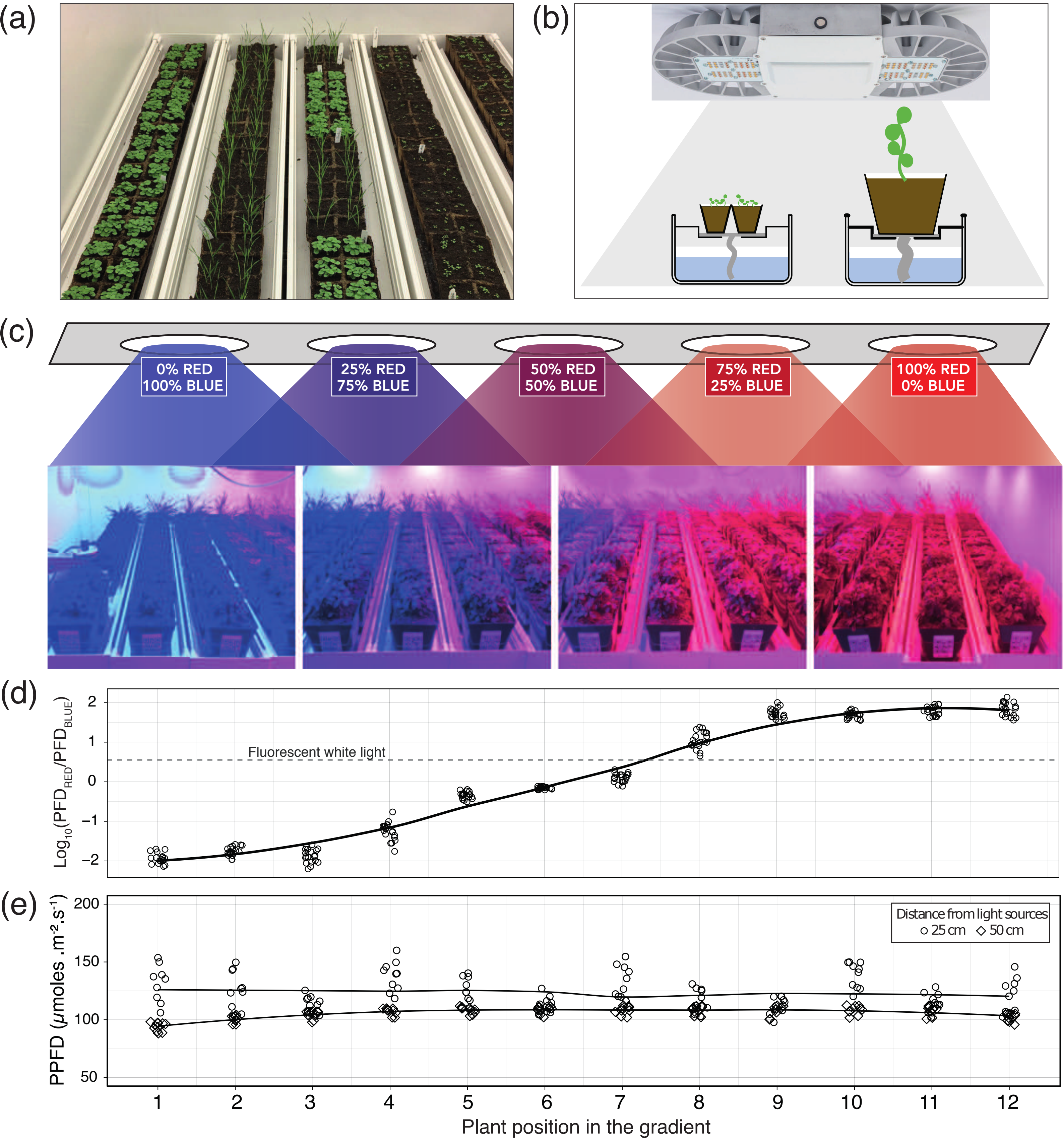

Figure2_PCA

Click here to access/download;Figure;Figure2_PCA.pdf

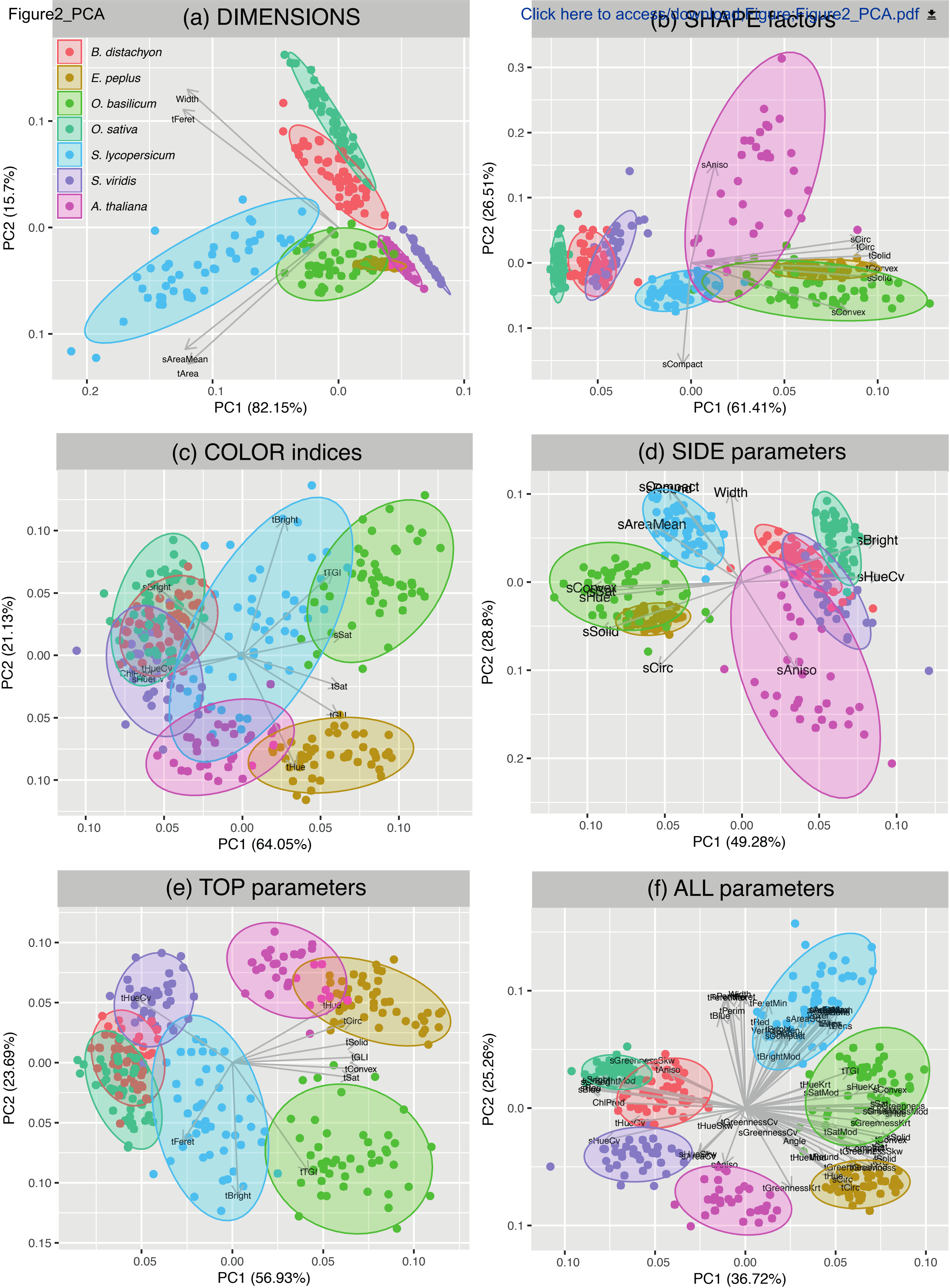

Figure3_Example_phenotype.pdf

Click here to access/download;Figure;Figure3_Example_phenotype.pdf

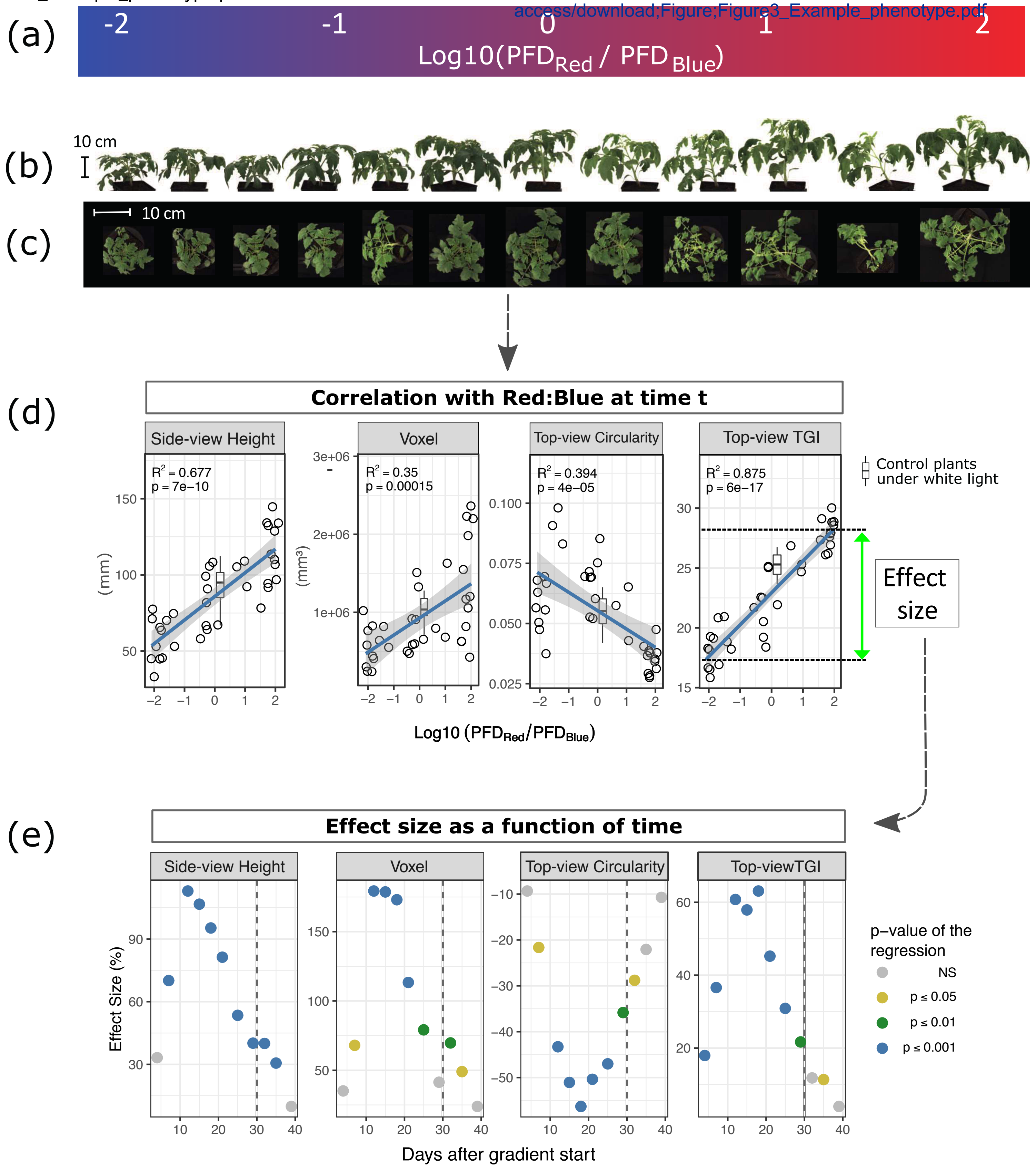

Figure4_Effect_size

Click here to access/download;Figure;Figure4_Effect-size.pdf

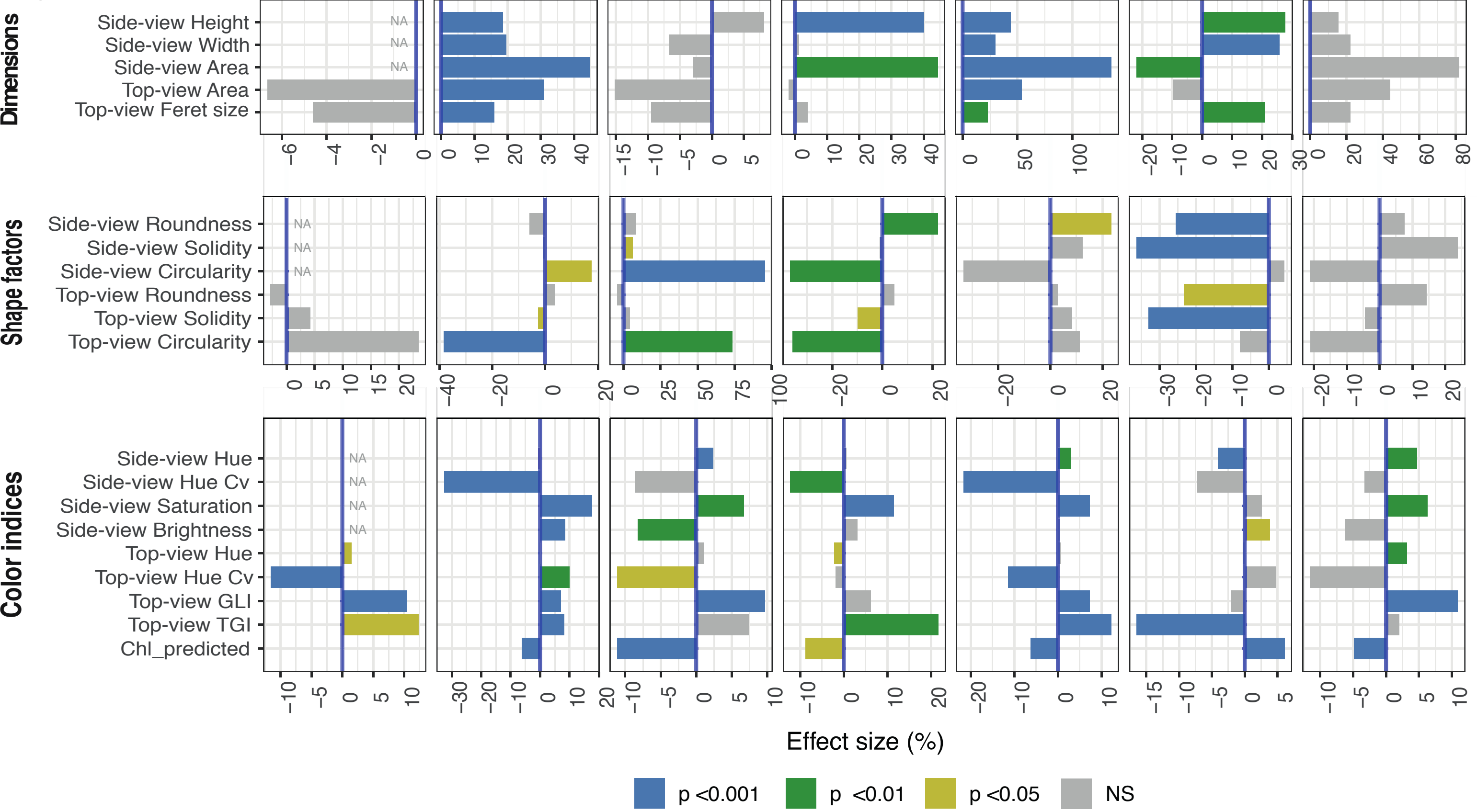

Figure5_Schematic-summary
Click here to access/download;Figure;Figure5_Schematic-summary.pdf

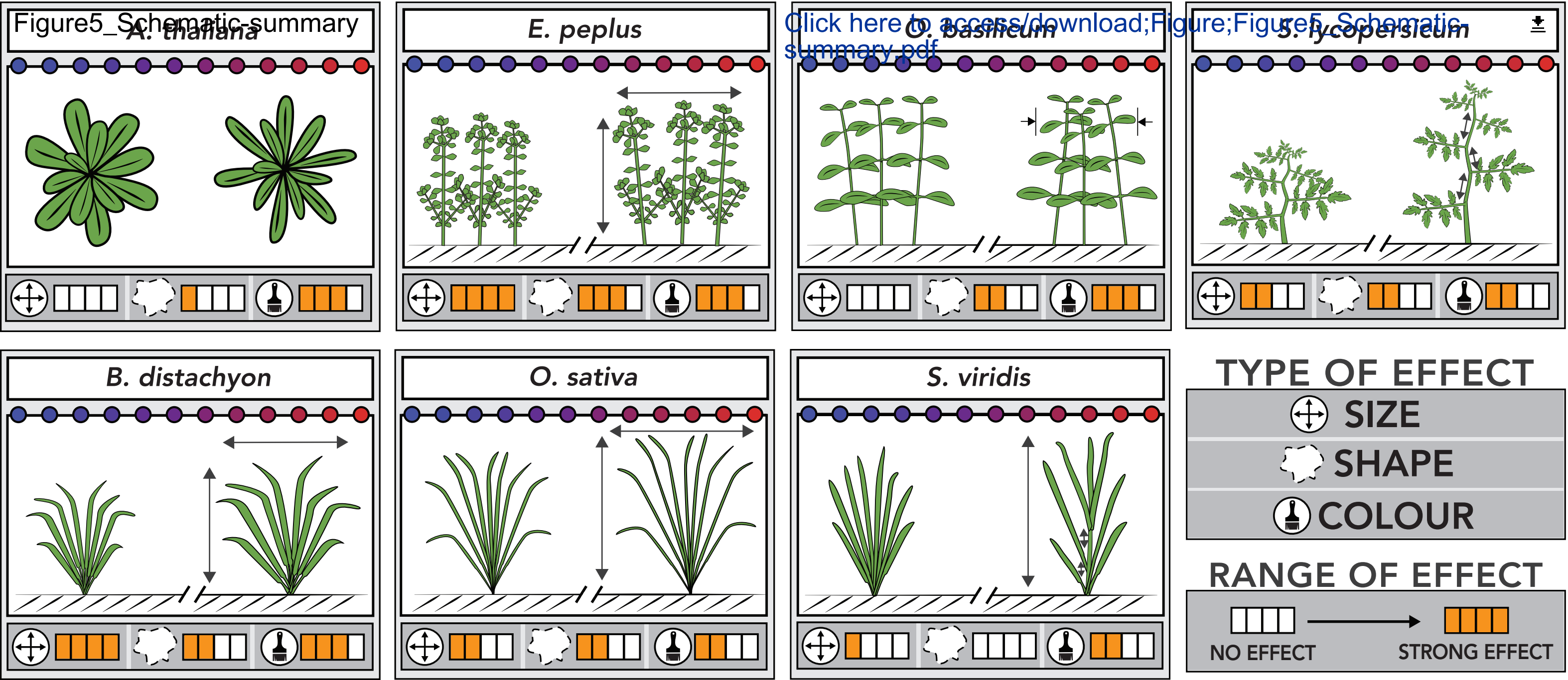

Click here to access/download;Figure;Figure6_Timecourses.pdf

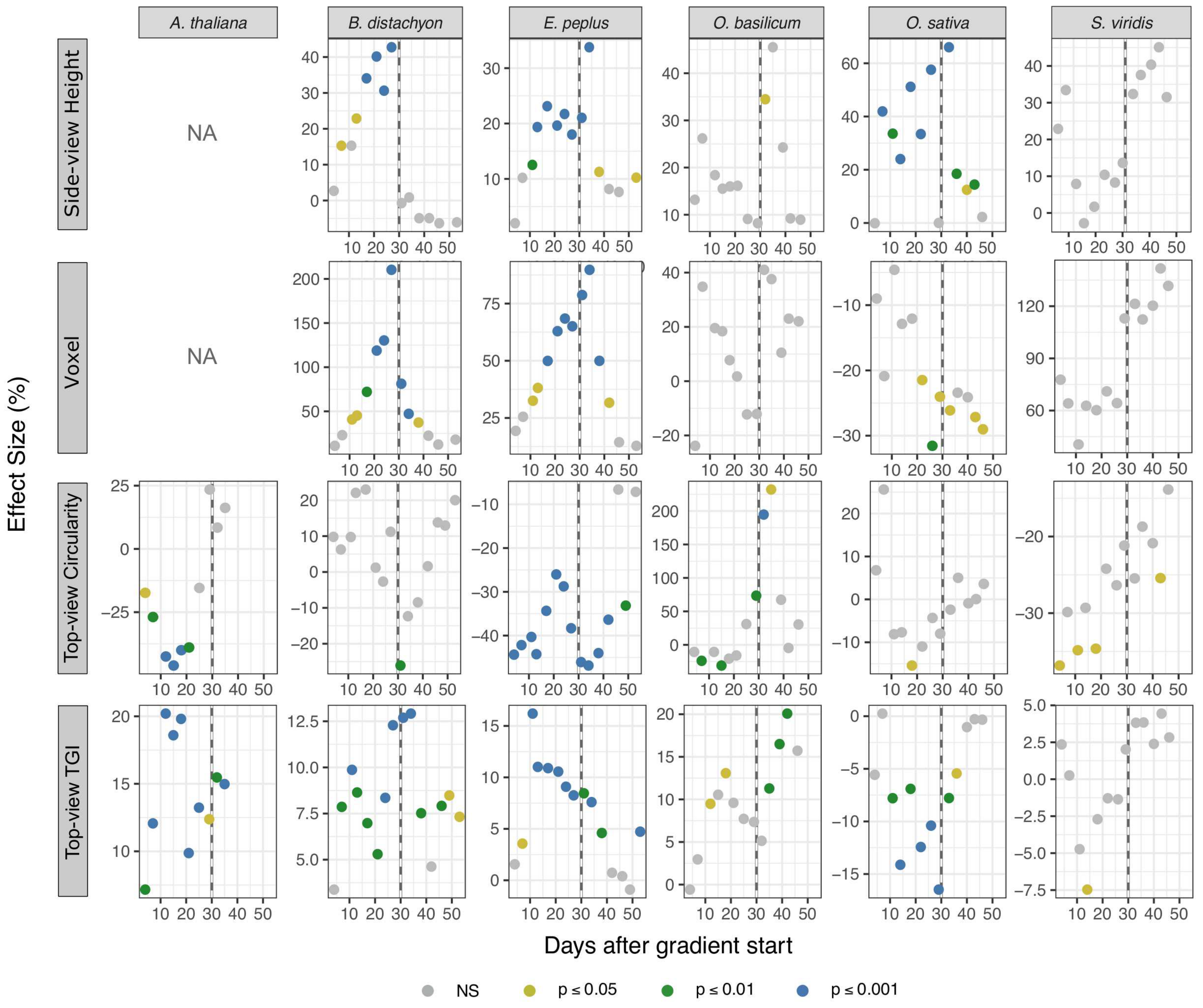

FigureS1_TGI-vs-Chl
Click here to access/download;Figure;FigureS1_TGI-vs-Chl.pdf

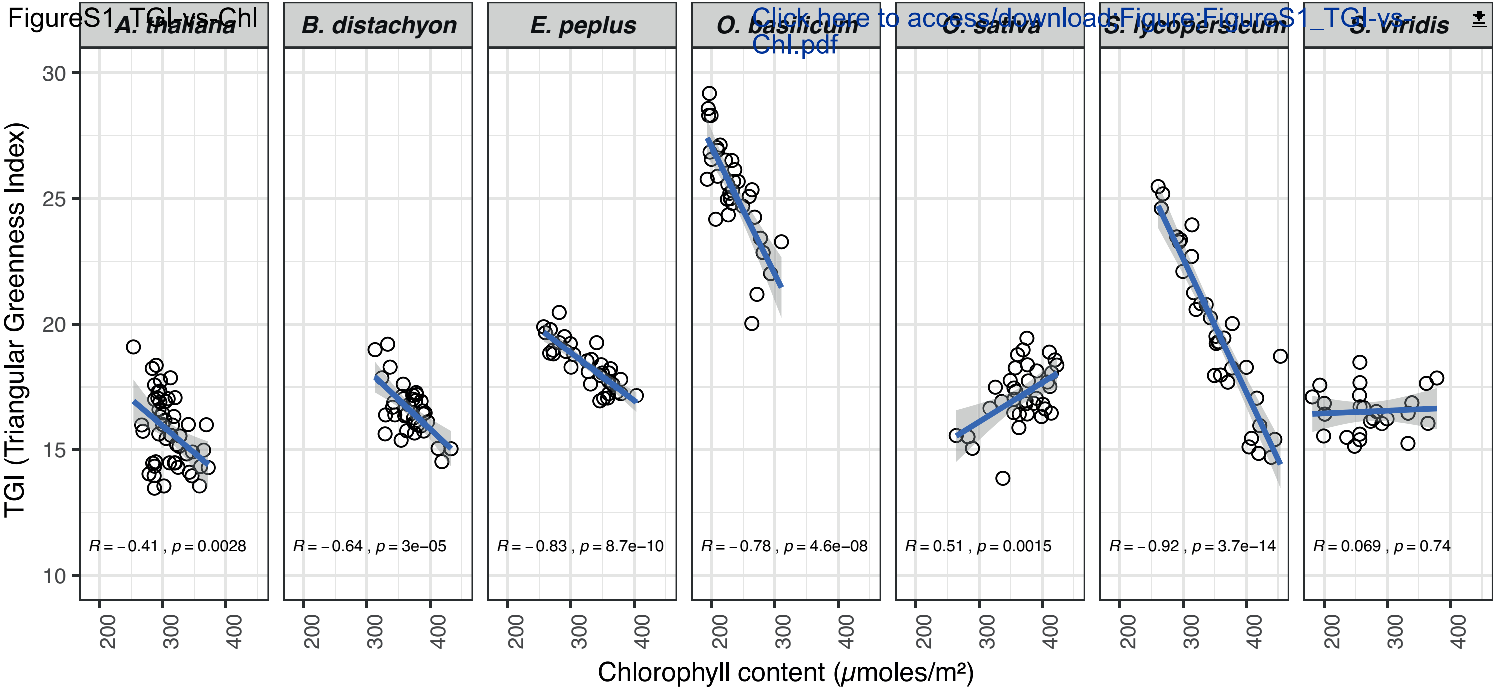

FigureS2_Imaging_blueprint
Click here to access/download;Figure;FigureS2_Imaging_b

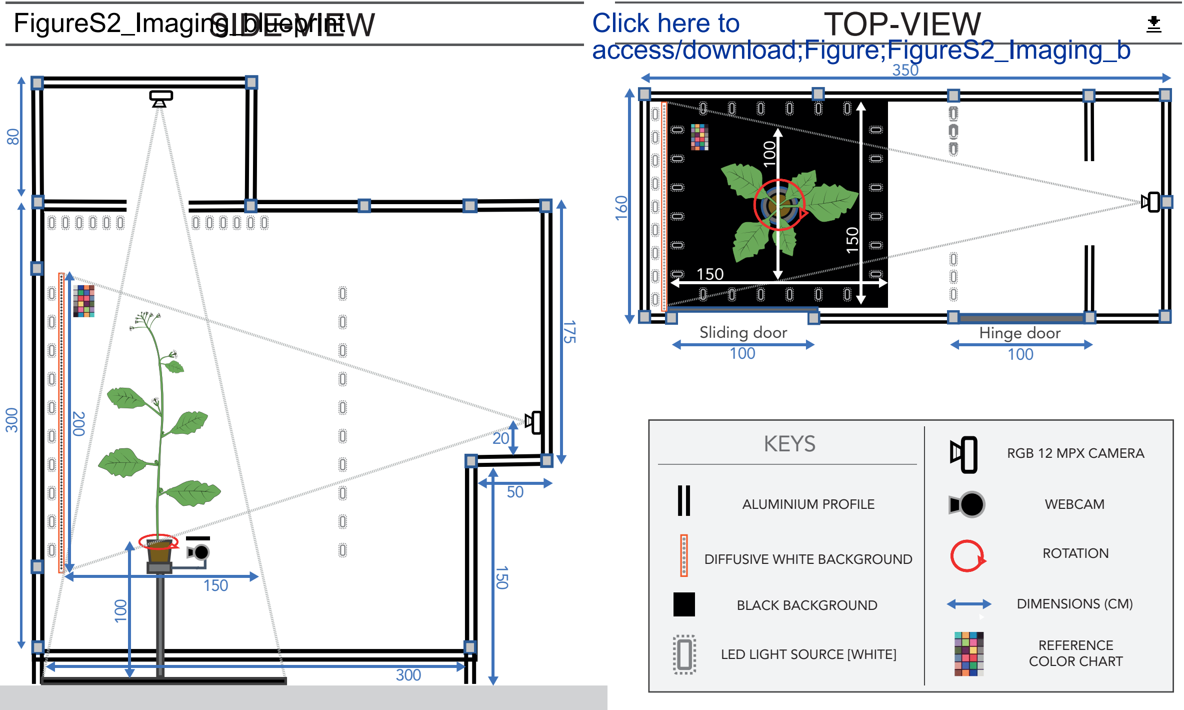

Supplement: giab101_GIGA-D-21-00184_Original_Submission [file giab101_giga-d-21-00184_original_submission.pdf]
