# Supplementary material for: LED color gradient as a new screening tool for rapid phenotyping of plant responses to light quality
Source: Gigascience. 2022 Jan 27;11:giab101. doi: 10.1093/gigascience/giab101 (PMC8848316; doi:10.1093/gigascience/giab101)
Supplement: giab101_Supplemental_Files [file giab101_supplemental_files.zip › FigureS1_Chamber layouts.pdf]

Chamber G9 - LED gradient - Sowing 1													
		Column											
		1	2	3	4	5	6	7	8	9	10	11	12
Row	1	1.1	1.2	1.3	1.4	1.5	1.6	1.7	1.8	1.9	1.10	1.11	1.12
	2	1.13	1.14	1.15	1.16	1.17	1.18	1.19	1.20	1.21	1.22	1.23	1.24
	3	1.25	1.26	1.27	1.28	1.29	1.30	1.31	1.32	1.33	1.34	1.35	1.36
	4	3.1	3.2	3.3	3.4	3.5	3.6	3.7	3.8	3.9	3.10	3.11	3.12
	5	3.13	3.14	3.15	3.16	3.17	3.18	3.19	3.20	3.21	3.22	3.23	3.24
	6	3.25	3.26	3.27	3.28	3.29	3.30	3.31	3.32	3.33	3.34	3.35	3.36
	7	3.37	3.38	3.39	3.40	3.41	3.42	3.43	3.44	3.45	3.46	3.47	3.48
	8	2.1	2.2	2.3	2.4	2.5	2.6	2.7	2.8	2.9	2.10	2.11	2.12
	9	2.13	2.14	2.15	2.16	2.17	2.18	2.19	2.20	2.21	2.22	2.23	2.24
	10	2.25	2.26	2.27	2.28	2.29	2.30	2.31	2.32	2.33	2.34	2.35	2.36

Chamber G4 - LED gradient - Sowing 3														
		Column												
		1	2	3	4	5	6	7	8	9	10	11	12	
Row	1	7.1	7.2	7.3	7.4	7.5	7.6	7.7	7.8	7.9	7.10	7.11	7.12	
	2	7.13	7.14	7.15	7.16	7.17	7.18	7.19	7.20	7.21	7.22	7.23	7.24	
	3	7.25	7.26	7.27	7.28	7.29	7.30	7.31	7.32	7.33	7.34	7.35	7.36	↑
	4	9.1	9.2	9.3	9.4	9.5	9.6	9.7	9.8	9.9	9.10	9.11	9.12	
	5	9.13	9.14	9.15	9.16	9.17	9.18	9.19	9.20	9.21	9.22	9.23	9.24	1.3m
	6	9.25	9.26	9.27	9.28	9.29	9.30	9.31	9.32	9.33	9.34	9.35	9.36	
	7	9.37	9.38	9.39	9.40	9.41	9.42	9.43	9.44	9.45	9.46	9.47	9.48	↓
	8	8.1	8.2	8.3	8.4	8.5	8.6	8.7	8.8	8.9	8.10	8.11	8.12	
	9	8.13	8.14	8.15	8.16	8.17	8.18	8.19	8.20	8.21	8.22	8.23	8.24	
	10	8.25	8.26	8.27	8.28	8.29	8.30	8.31	8.32	8.33	8.34	8.35	8.36	
		← 2.3m →												

Chamber G5 - LED gradient - Sowing 2													
		Column											
		1	2	3	4	5	6	7	8	9	10	11	12
Row	1	4.1	4.2	4.3	4.4	4.5	4.6	4.7	4.8	4.9	4.10	4.11	4.12
	2	4.13	4.14	4.15	4.16	4.17	4.18	4.19	4.20	4.21	4.22	4.23	4.24
	3	4.25	4.26	4.27	4.28	4.29	4.30	4.31	4.32	4.33	4.34	4.35	4.36
	4	6.1	6.2	6.3	6.4	6.5	6.6	6.7	6.8	6.9	6.10	6.11	6.12
	5	6.13	6.14	6.15	6.16	6.17	6.18	6.19	6.20	6.21	6.22	6.23	6.24
	6	6.25	6.26	6.27	6.28	6.29	6.30	6.31	6.32	6.33	6.34	6.35	6.36
	7	6.37	6.38	6.39	6.40	6.41	6.42	6.43	6.44	6.45	6.46	6.47	6.48
	8	5.1	5.2	5.3	5.4	5.5	5.6	5.7	5.8	5.9	5.10	5.11	5.12
	9	5.13	5.14	5.15	5.16	5.17	5.18	5.19	5.20	5.21	5.22	5.23	5.24
	10	5.25	5.26	5.27	5.28	5.29	5.30	5.31	5.32	5.33	5.34	5.35	5.36

Chamber G8 - White light - Sowings 1, 2, 3													
		Column											
		1	2	3	4	5	6	7	8	9	10	11	12
Row	1	10.1	10.2	12.5	12.6	14.1	14.2	13.9	.13.10	18.1	18.2		
	2	10.3	10.4	12.7	12.8	14.3	14.4	.13.11	.13.12	18.3	18.4		
	3	11.1	11.2	10.9	.10.10	15.1	15.2	14.9	14.10	16.5	16.6	17.9	
	4	11.3	11.4	.10.11	.10.12	15.3	15.4	14.11	14.12	16.7	16.8	17.11	
	5	12.1	12.2	11.9	1.10	13.5	13.6	15.9	15.10	17.5	17.6	18.9	
	6	12.3	12.4	11.11	11.12	13.7	13.8	15.11	15.12	17.7	17.8	18.11	
	7	10.5	10.6	12.9	12.10	14.5	14.6	16.1	16.2	18.5	18.6	17.10	
	8	10.7	10.8	12.11	12.12	14.7	14.8	16.3	16.4	18.7	18.8	17.12	
	9	11.5	11.6	13.1	13.2	15.5	15.6	17.1	17.2	16.9	16.10	18.10	
	10	11.7	11.8	13.3	13.4	15.7	15.8	17.3	17.4	16.11	16.12	18.12	

S. lycopersicum · *A. thaliana* · *O. basilicum* · *B. distachyon* · *E. peplus* · *O. sativa* · *S. viridis*
