# Supplementary material for: LED color gradient as a new screening tool for rapid phenotyping of plant responses to light quality
Source: Gigascience. 2022 Jan 27;11:giab101. doi: 10.1093/gigascience/giab101 (PMC8848316; doi:10.1093/gigascience/giab101)
Supplement: giab101_Supplemental_Files [file giab101_supplemental_files.zip › FigureS4_Imaging_blueprint.pdf]

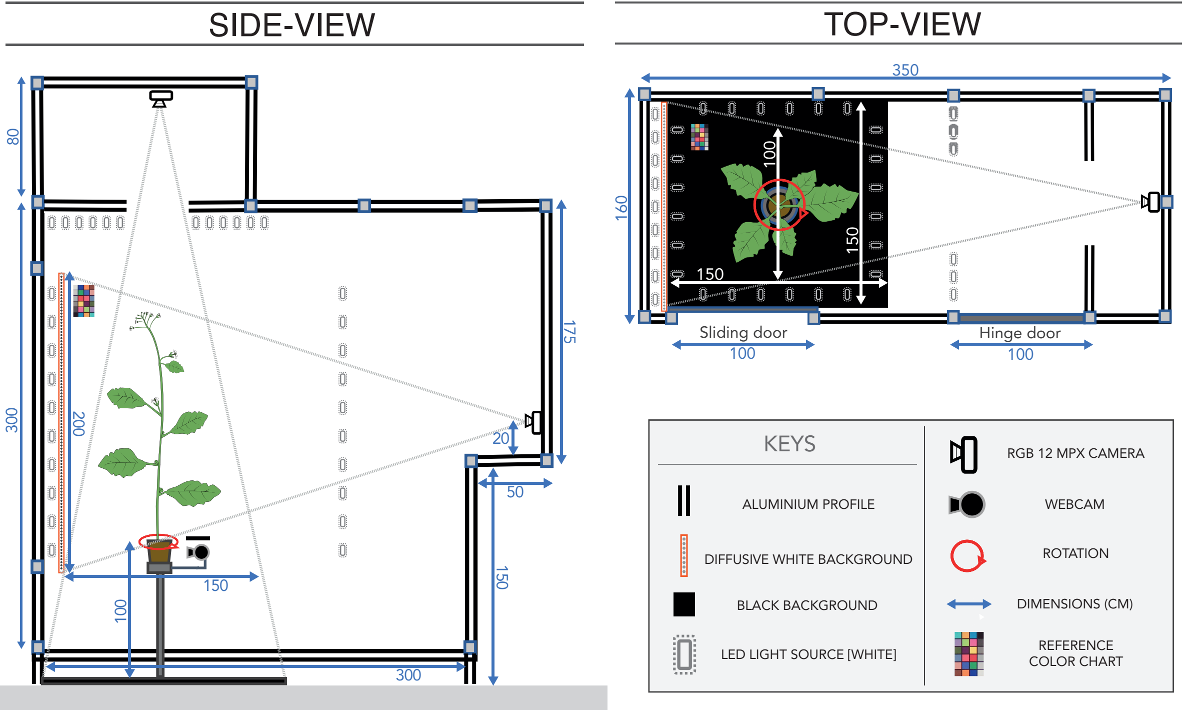

SIDE-VIEW
80
300
200
20
175
50
150
100
150
300
TOP-VIEW
350
160
100
150
150
Sliding door
100
Hinge door
100
KEYS
ALUMINIUM PROFILE
DIFFUSIVE WHITE BACKGROUND
BLACK BACKGROUND
LED LIGHT SOURCE [WHITE]
RGB 12 MPX CAMERA
WEBCAM
ROTATION
DIMENSIONS (CM)
REFERENCE COLOR CHART
