# Supplementary figures and images for: LED color gradient as a new screening tool for rapid phenotyping of plant responses to light quality
Source: Gigascience. 2022 Jan 27;11:giab101. doi: 10.1093/gigascience/giab101 (PMC8848316; doi:10.1093/gigascience/giab101)

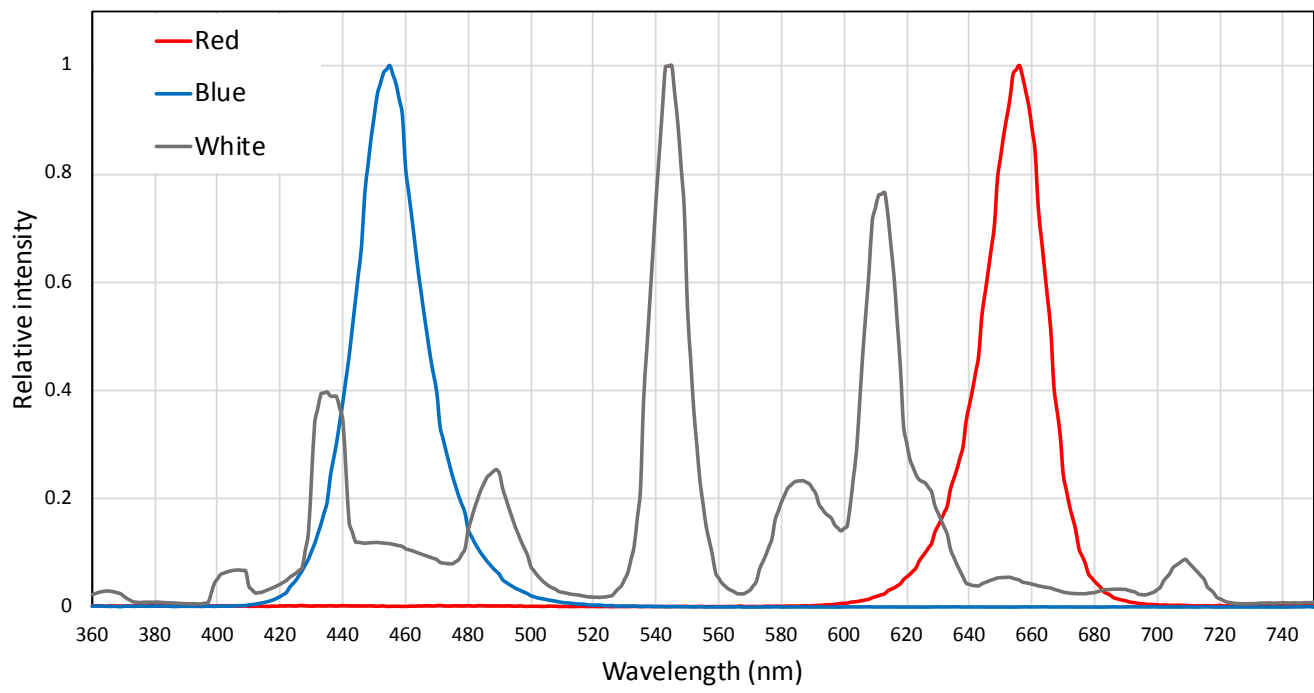

Supplement: giab101_Supplemental_Files [file giab101_supplemental_files.zip › FigureS2_Red_Blue_White_Spectra.pdf]

TGI (Triangular Greenness Index)

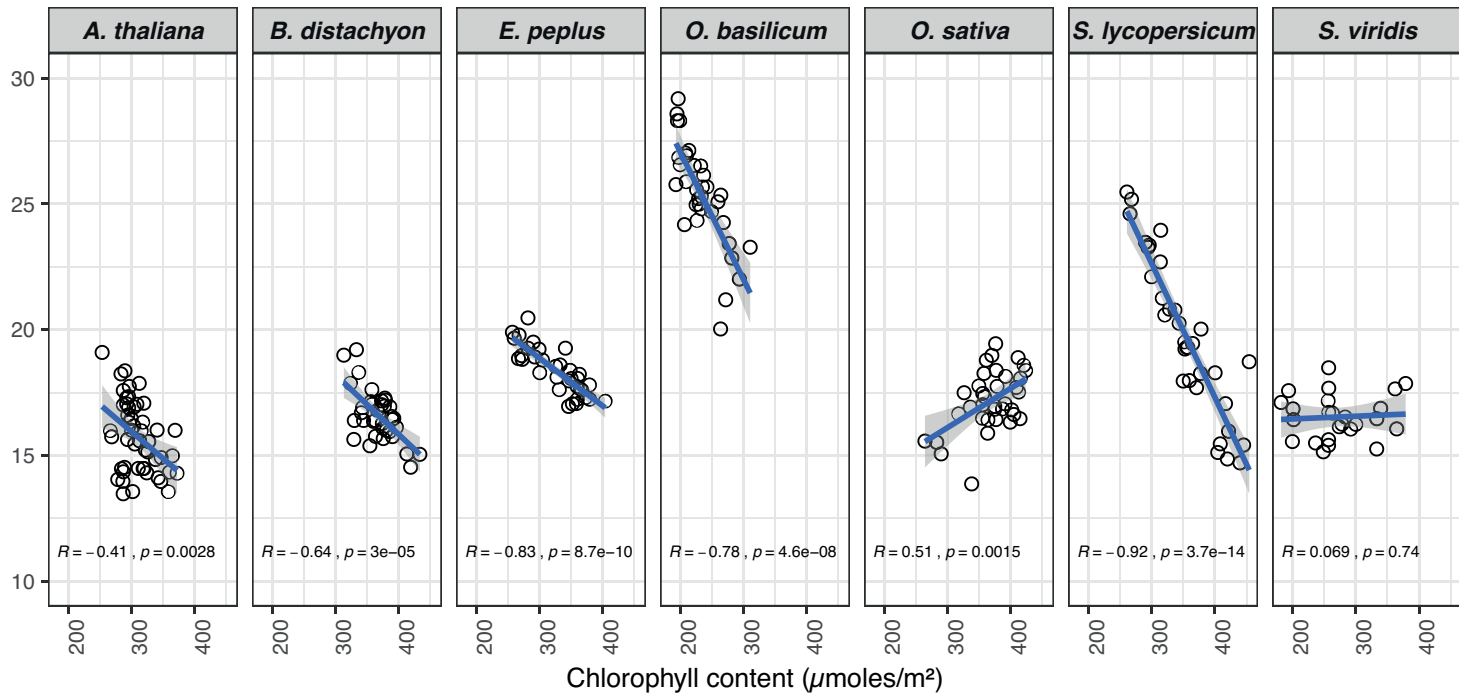

Supplement: giab101_Supplemental_Files [file giab101_supplemental_files.zip › FigureS3_TGI-vs-ChI.pdf]
