# Supplementary material for: LED color gradient as a new screening tool for rapid phenotyping of plant responses to light quality
Source: Gigascience. 2022 Jan 27;11:giab101. doi: 10.1093/gigascience/giab101 (PMC8848316; doi:10.1093/gigascience/giab101)
Supplement: giab101_Response_to_Reviewer_Comments_Original_Submission [file giab101_response_to_reviewer_comments_original_submission.pdf]

Author's Response To Reviewer Comments

Close

Dear Editor,
Dear Reviewers,

First of all, on behalves of all co-authors, I would like to thank you for handing our manuscript, for your positive comments and suggestions.
Below we have addressed all concerns and replied point-by-point in your reports.
We hope that you will be satisfied by our responses and by the changes we have integrated in the revised submission.

We thank you in advance and are looking forward to receiving your feedback.

Sincerely yours,

Claire Périlleux

Reviewer #1:
The manuscript presents the result of an experiment investigating the impact of red:blue ratio of light gradient on plant phenotypic traits in seven plant species. The subject of the manuscript is very innovative and interesting, but there are parts of the materials and methods that are less clear.

Specific comments:
* In this study, plant phenotypic traits were evaluated using an imaging platform. Plant biomass (fresh and dry weights of shoot and root) is one of the most important plant growth parameters. Are there any suggestions that plant biomass can be predicted from the plant phenotypic traits quantified by the imaging platform?

Answer: The prediction of plant biomass from non-destructive image descriptors is a question that is frequently addressed in the literature, and several examples can be found where a correlative model was built and used. We have added relevant references in the text (page4, lines 6-8). Our experiment did not include destructive sampling for weighing because our priority was to perform a time course phenotyping in order to capture subtle effects of the Red:Blue ratio.

* Growth conditions:
* Does the irradiance of 130-150 µE·m-2·s-1 indicate the PPFD (400-700 nm)? How was it measured?

Answer: Yes, it is PPFD, and it was measured using the spectrophotometer mentioned in section "Red-Blue light gradient". The text has been edited accordingly.

* Please be consistent for the unit for photon flux density throughout the manuscript. µEinsteins were interchangeably used along with µmol·m-2·s-1 in the past, but the Einstein is not a unit in the SI of units. Thus, please use µmol·m-2·s-1 when you quantify the photon flux density. Also, please revise the µmoles·m-2·s-1 in Fig. 1 into µmol·m-2·s-1.

Answer: We agree with the reviewer's comment and this issue is now addressed.

* Could you provide the spectral distribution data for white light, red LED, and blue LED used in this study?

Answer: We have added a supplemental figure (Figure S2) that provides the spectral distribution for white fluorescent lights, as well as blue and red LED used in this study. This figure is referenced in the

M&M section.

* For the concentration of the slow release fertilizer, do you mean gram per liter? If so, please correct it to 6 g·L-1.

Answer: Yes, indeed. This issue has been corrected.

* What was the growing conditions (air temperature, relative humidity, photoperiod, etc) during the treatment of red:blue gradient?

Answer: The following sentence was added: "The other parameters used during the gradient treatment (air temperature, relative humidity, photoperiod) were the same as described in the "Growth conditions" section."

* Did you keep the control plants under white light continuously? Then, did you make sure that the control plants and treatment plants are grown under the same growing condition except for the light quality treatment?

Answer: The plants exposed to white light and Red:Blue gradient were grown in neighboring cabinets having exactly the same design, regulation performance, and parametrization. This being said, we wish to clarify that plants grown under white light were not "control" plants but rather a "reference" to traditional practice, since our aim was to characterize the effect of the Red:Blue ratio across the gradient and not in comparison to white light.

* It is not clear whether the experiment was replicated. The experimental unit is the physical entity which can be assigned, at random, to a treatment. Here the experiment unit was the experimental plot under each light gradient treatment. A single plant should be treated as an observational unit. So, without replications, the data is less reliable.

Answer: The intentions with this experiment were primarily to obtain a proof of concept for using color light gradients versus discrete conditions, and to explore how the data could be analyzed and visualized in an efficient and useful way. With that in mind, different species were included to test how the setup would work for different plant growth habits / morphologies and to increase the chance of detecting phenotypic changes in such exploratory experiment. From a cost/capacity point of view, we do not believe that repeating the complete experiment is essential to reinforce the proof of concept, but we absolutely agree that more replication would be useful if the goal were to formally establish biological responses. In that respect, the experiment presented here can be a good basis for designing more specific experiments requiring higher replication.

Reviewer #2:
The authors used imaging tools with three types of phenotypic descriptors (dimensions, shape, colour indices) and side- or top-camera views in order to determine non-destructive parameters of seven diverse species (Arabidopsis thaliana, Brachypodium distachyon, Euphorbia peplus, Ocimum basilicum, Oryza sativa, Solanum lycopersicum, and Setaria viridis) growing under different Red/Blue gradients (from 100% Blue to 100% Red). The results are important since they are non-destructive and provide a good basis for the selection of light treatments for specific plants in controlled environment agriculture. The introduction is informative and sufficiently describes the scope of the research. I like the way the authors describe/display the results. Relatively few words (compared to the volume of the obtained measurements) but beautifully built figures which provide all the necessary information.

However, I would expect more discussion at the end of each set of parameters
results' description, as well as possible comparisons with the literature, even if it is rather scarce. For example, in PDF page 11, subsection "Patterns of change over time", the results are barely discussed.

Answer: We have included more discussion and references to the literature in the "Results" section that is now entitled "Results and Discussion", especially between p8 line15 and p9 line15, between p9 line 27 and p10 line 5, between page 10 line 22 and page 11 line 4. We have paid attention, however, not to "overwhelm" the readers with details that are usually available for model plants such as Arabidopsis and rice, but not for the other species used in this study.

Moreover, the review process would be facilitated if the manuscript had line numbering.

Answer : We have added line numbering in the revised manuscript.

Specific comments are following:
* In the title, LED should be written with capital letters, not Led

Answer: Yes indeed, this has been corrected.

* Keywords must not be included in the title. Please remove or substitute LED and light quality
Answer: We removed these terms from keywords.

Introduction
* PDF page 4, L3. Controlled environment agriculture must be abbreviated the first time it is written in the text. The same applies with other terms such as RGB.

Answer : CEA, RGB, QR-code, and UID abbreviations are now defined and subsequently used in the main text.

* PDF page 5, L23. "Large-scale crops" is a more appropriate term.
Answer: The term was changed.

* I agree with the active voice in the objectives' part of the introduction. However, you should refrain from beginning most sentences with "we".
Answer : We agree with the reviewer’s comment and the style was adapted.

Results
* PDF page 8. I suggest that "Data description" subsection is moved in the "Methods" Section

Answer : It is our understanding that, in GigaScience papers, the “Data description” section should stand on its own, because it is a data-science oriented journal, and so we followed the standard layout as requested in the instructions to authors.
We suggest letting the Editor decide on this point.

* This section should be renamed to "Results and Discussion" since there is also discussion within the results.

Answer : We have renamed this section and hope that the Editor agrees. We thought indeed that it was useful to have some discussion within the results rather than in a separate section.

Methods
* PDF page 14. How many cabinets were used? How many treatments and plants were placed in each cabinet? Apart from figure 1 depiction, you should also describe the experimental design in order for the reader (and me as well) to fully understand it.

Answer : To clarify these points, we have added the supplemental Figure S3 that shows the experimental design and layout during the gradient treatment, with additional details in the caption.

Close
