# Supplementary material for: LED color gradient as a new screening tool for rapid phenotyping of plant responses to light quality
Source: Gigascience. 2022 Jan 27;11:giab101. doi: 10.1093/gigascience/giab101 (PMC8848316; doi:10.1093/gigascience/giab101)
Supplement: giab101_Reviewer_2_Report_Original_Submission — Filippos Bantis -- 8/16/2021 Reviewed [file giab101_reviewer_2_report_original_submission.pdf]

Reviewer Report

Title: LED Color Gradient As A New Screening Tool For Rapid Phenotyping Of Plant Responses To Light Quality

Version: Original Submission **Date:** 8/16/2021

Reviewer name: Filippos Bantis

Reviewer Comments to Author:

The authors used imaging tools with three types of phenotypic descriptors (dimensions, shape, colour indices) and side- or top-camera views in order to determine non-destructive parameters of seven diverse species (Arabidopsis thaliana, Brachypodium distachyon, Euphorbia peplus, Ocimum basilicum, Oryza sativa, Solanum lycopersicum, and Setaria viridis) growing under different Red/Blue gradients (from 100% Blue to 100% Red). The results are important since they are non-destructive and provide a good basis for the selection of light treatments for specific plants in controlled environment agriculture. The introduction is informative and sufficiently describes the scope of the research. I like the way the authors describe/display the results. Relatively few words (compared to the volume of the obtained measurements) but beautifully built figures which provide all the necessary information. However, I would expect more discussion at the end of each set of parameters results' description, as well as possible comparisons with the literature, even if it is rather scarce. For example, in PDF page 11, subsection "Patterns of change over time", the results are barely discussed. Moreover, the review process would be facilitated if the manuscript had line numbering.

Specific comments are following:

* In the title, LED should be written with capital letters, not Led
* Keywords must not be included in the title. Please remove or substitute LED and light quality

Introduction

* PDF page 4, L3. Controlled environment agriculture must be abbreviated the first time it is written in the text. The same applies with other terms such as RGB.
* PDF page 5, L23. "Large-scale crops" is more appropriate term.
* I agree with the active voice in the objectives' part of the introduction. However, you should refrain from beginning most sentences with "we".

Results

* PDF page 8. I suggest that "Data description" subsection is moved in the "Methods" section
* This section should be renamed to "Results and Discussion" since there is also discussion within the results.

Methods

* PDF page 14. How many cabinets were used? How many treatments and plants were placed in each cabinet? Apart from figure 1 depiction, you should also describe the experimental design in order for the reader (and me as well) to fully understand it.

Methods

Are the methods appropriate to the aims of the study, are they well described, and are necessary controls included? Choose an item.

Conclusions

Are the conclusions adequately supported by the data shown? Choose an item.

Reporting Standards

Does the manuscript adhere to the journal's guidelines on minimum standards of reporting? Choose an item.

Choose an item.

Statistics

Are you able to assess all statistics in the manuscript, including the appropriateness of statistical tests used? Choose an item.

Quality of Written English

Please indicate the quality of language in the manuscript: Choose an item.

Declaration of Competing Interests

Please complete a declaration of competing interests, considering the following questions:

- Have you in the past five years received reimbursements, fees, funding, or salary from an organisation that may in any way gain or lose financially from the publication of this manuscript, either now or in the future?
- Do you hold any stocks or shares in an organisation that may in any way gain or lose financially from the publication of this manuscript, either now or in the future?
- Do you hold or are you currently applying for any patents relating to the content of the manuscript?
- Have you received reimbursements, fees, funding, or salary from an organization that holds or has applied for patents relating to the content of the manuscript?
- Do you have any other financial competing interests?
- Do you have any non-financial competing interests in relation to this paper?

If you can answer no to all of the above, write 'I declare that I have no competing interests' below. If your reply is yes to any, please give details below.

I declare that I have no competing interests

I agree to the open peer review policy of the journal. I understand that my name will be included on my report to the authors and, if the manuscript is accepted for publication, my named report including any attachments I upload will be posted on the website along with the authors' responses. I agree for my report to be made available under an Open Access Creative Commons CC-BY license (http://creativecommons.org/licenses/by/4.0/). I understand that any comments which I do not wish to be included in my named report can be included as confidential comments to the editors, which will not be published.

Choose an item.

To further support our reviewers, we have joined with Publons, where you can gain additional credit to further highlight your hard work (see: https://publons.com/journal/530/gigascience). On publication of this paper, your review will be automatically added to Publons, you can then choose whether or not to claim your Publons credit. I understand this statement.

Yes Choose an item.
